# Maintenance of Sex-Related Genes and the Co-Occurrence of Both Mating Types in *Verticillium dahliae*

**DOI:** 10.1371/journal.pone.0112145

**Published:** 2014-11-10

**Authors:** Dylan P. G. Short, Suraj Gurung, Xiaoping Hu, Patrik Inderbitzin, Krishna V. Subbarao

**Affiliations:** 1 Department of Plant Pathology, University of California Davis, Salinas, CA, United States of America; 2 State Key Laboratory of Crop Stress Biology for Arid Areas and College of Plant Protection, Northwest A&F University, Yangling, Shaanxi, China; Georg-August-University of Göttingen Institute of Microbiology & Genetics, Germany

## Abstract

*Verticillium dahliae* is a cosmopolitan, soilborne fungus that causes a significant wilt disease on a wide variety of plant hosts including economically important crops, ornamentals, and timber species. Clonal expansion through asexual reproduction plays a vital role in recurring plant epidemics caused by this pathogen. The recent discovery of recombination between clonal lineages and preliminary investigations of the meiotic gene inventory of *V. dahliae* suggest that cryptic sex appears to be rare in this species. Here we expanded on previous findings on the sexual nature of *V. dahliae*. Only 1% of isolates in a global collection of 1120 phytopathogenic *V. dahliae* isolates contained the *MAT1-1* idiomorph, whereas 99% contained *MAT1-2*. Nine unique multilocus microsatellite types comprised isolates of both mating types, eight of which were collected from the same substrate at the same time. Orthologs of 88 previously characterized sex-related genes from fungal model systems in the Ascoymycota were identified in the genome of *V*. *dahliae*, out of 93 genes investigated. Results of RT-PCR experiments using both mating types revealed that 10 arbitrarily chosen sex-related genes, including *MAT1-1-1* and *MAT1-2-1*, were constitutively expressed in *V*. *dahliae* cultures grown under laboratory conditions. Ratios of non-synonymous (amino-acid altering) to synonymous (silent) substitutions in *V*. *dahliae MAT1-1-1* and *MAT1-2-1* sequences were indistinguishable from the ratios observed in the *MAT* genes of sexual fungi in the *Pezizomycotina*. Patterns consistent with strong purifying selection were also observed in 18 other arbitrarily chosen *V*. *dahliae* sex-related genes, relative to the patterns in orthologs from fungi with known sexual stages. This study builds upon recent findings from other laboratories and mounts further evidence for an ancestral or cryptic sexual stage in *V. dahliae*.

## Introduction

Sexual reproduction is thought [1] to act as a mechanism to combine fit alleles from different individuals, and to break apart locally disadvantageous allele combinations under dynamic selection pressures [2]. While sexual reproduction may in theory be costly and disrupt favorable gene combinations, experimental evidence has suggested that sex in fungi increases the rate of adaptation to new environments [3]. Prior to molecular techniques, the formation of sexual structures and spores was the primary evidence of sex in fungi. It is now evident that sex in many taxa is rare, unpredictable and elusive. For many fungi, the only documented sexual structures are formed on certain media and/or growth conditions *in vitro* [4], [5]. Some putatively asexual plant pathogens have been found to sexually reproduce in nature only in specific ecological conditions and geographic locales, such as near the center of origin of the species [6].

Advances in genetic markers and population biology have led to significant advances in the discovery of rare or cryptic sexual stages in fungi [7]. Populations of many species that lack obvious sexual stages in nature nevertheless have been found to harbor molecular patterns of sexuality based on investigations of mating type frequencies, population structure, multilocus linkage disequilibrium [8], [9] and computer simulations [6]. Additionally, bioinformatic surveys of complete genomes, have been used to infer sexuality based on the meiotic gene inventory [10]–[13]. Advances in genomics have enabled the unprecedented implementation of these approaches to investigate sexuality in fungi. Many seemingly asexual fungi have retained the genes required for the sexual “machinery”, including many that are important to the fields of agriculture and medicine [12], [14]–[16].

*Verticillium* is a small genus of phytopathogenic fungi that causes billions of dollars in agricultural losses annually [17]. *Verticillium dahliae* is a cosmopolitan, soilborne plant pathogen that causes an economically significant wilt disease. It is known for its extremely wide host range [18] and its ability to survive in soils as dormant resting structures for many years [17], [19]. Historically, *V. dahliae* has been considered strictly asexual because it has failed to form sexual structures under the laboratory conditions tested. Vegetative anastomosis, the fusion of growing hyphae under laboratory conditions, has been reported [20], [21], and several vegetative compatibility groups (VCGs) have been classified. Deep sequencing of all known VCGs of *V. dahliae* has revealed that VCGs are strongly correlated to clonal lineages [22], but has also revealed that putative sexual recombination between clonal lineages has occurred rarely [23].

Sexual compatibility and fruiting body formation in heterothallic fungi in the Ascoymycota is determined by a variety of sex-related gene pathways. Of primary importance are the two idiomorphs of the *MAT* locus, which differ in gene content and are the master regulators of sexual recombination in the Ascoymycota [24]. One idiomorph contains a critical gene that encodes an α domain (*MAT1-1-1*), while the other contains a critical gene that encodes a DNA-binding domain of the high-mobility group (HMG) type (*MAT1-2-1*) [25]. Isolates with either of the idiomorphs are referred to as *MAT1-1* or *MAT1-2* [26]. *Verticillium dahliae* is considered heterothallic because both idiomorphs are known to exist [27], and only one idiomorph has been observed in any one isolate.

Previous sequences of the α and HMG domains of *V*. *dahliae MAT* genes showed high amino acid conservation with other fungi in the subphylum *Pezizomycotina* [27], [28]. Mating type frequencies in *V*. *dahliae* have been reported in multiple studies as skewed [23], [28], [29]. Although previous studies have reported mating type distributions skewed towards *MAT1-2* in *V. dahliae*, they have not clearly stated whether both mating types are sympatric in nature, that is, whether isolates of opposite mating coexist in nature. It is also unknown whether genetically identical multilocus microsatellite types contain both *MAT* idiomorphs, a condition which has previously been interpreted as unequivocal evidence for sexual recombination [11].

In addition to the presence of both mating types, other molecular signatures suggestive of sex have been reported in *V. dahliae*. Multilocus linkage equilibrium has been reported in collections of *V. dahliae* [30], although clonal expansion is of primary importance in pathogen reproduction and dissemination within regions where this pathogen is a severe problem in agriculture [31]. However, even in species with known sexual stages, signatures of clonality can predominate in multilocus data sets [32]. Gene trees with incongruent topologies may be a robust indicator of meiotic recombination when they occur within a strongly supported phylogenetic species [33], [34]. Gene trees with incongruent topologies were previously reported in *V. dahliae* based on sequences of the protein coding genes *actin* (*ACT*), *elongation factor 1-alpha* (*EF*), *glyceraldehyde-3-phosphate dehydrogenase* (*GPD*), and *tryptophan synthase* (*TS)* [35]. The strongest evidence yet of recombination between lineages of *V. dahliae* was based on over 20,000 single nucleotide polymorphisms (SNPs) [23].

Genomic investigations of *V. dahliae* have also provided some evidence of sexuality in *V. dahliae.* For example, a single homolog of the gene encoding the DNA methyltranferase (DMT) *RID* exists in *V. dahliae* reference strain Ls 17, a gene which was first characterized as part of the Repeat-Induced Point (RIP) machinery in *N. crassa* [36]. Patterns consistent with RIP-like mutation were subsequently discovered in the *V. dahliae* genome in multiple long interspersed element (LINE)-like and long terminal repeat (LTR) retroelement sequences [37] and other transposons [38]. Furthermore, preliminary explorations of the meiotic gene inventory have revealed the presence of genes known to function in sex-related pathways in other fungal systems [23].

Comparative population genomics of *V. dahliae* has significantly advanced the understanding of the molecular basis of races, as well as the existence of inter-Kingdom horizontal gene transfer [39], and has also led some researchers to posit chromosomal reshuffling (genomic rearrangements and chromosomal length polymorphisms, despite a high degree of sequence conservation) as the sole mechanism for generating the diversity observed within *V. dahliae* [40]. Significant chromosomal rearrangements are expected to interfere with meiosis [41], so it is reasonable to expect sex to be impossible between isolates with extreme karyotypic polymorphisms [40], .

It has been postulated that a detailed understanding of the genes required for the initiation and completion of meiosis in sexual fungi, that it should be possible to understand the molecular mechanisms that control sexual compatibility and to determine which of these genes are missing or nonfunctional in asexual fungi [43]. In fact, imperfect functioning of mating type genes and other sexual factors such as pheromone receptors have been hypothesized in *V. dahliae* [27]. In the context of exploring the functionality of sex-related genes (and not merely the existence of pseudogenes), Reverse transcriptase-PCR has been used to show that both mating type genes are expressed in fungi, for which no known sexual stage has been documented [44], while other studies have demonstrated pheromone receptor and precursor gene expression in other putatively asexual fungi [45]. To date, the expression of *MAT* genes and other sex-related genes in *V. dahliae* has never been investigated.

Evolutionary theory predicts that if amino acid-altering genetic mutations occur in genes or domains of critical function and result in lower fitness, they will be purged from populations through purifying selection [46]. Conversely, selection acting on mutations in non-essential genes or domains is “relaxed”, and thus accumulation of amino acid-altering mutations is more likely in such regions. Calculations of the Ka/Ks ratios in a set of amino acid sequences can thus be used to estimate an evolutionary history of both positive and purifying selection at each amino-acid site. Strong purifying selection in 9,471 core eukaryotic genes was previously reported in the genomes of several isolates of *V. dahliae* [40]. Whether sex-related genes in the *V. dahliae* genome are similarly conserved, compared to related sexual fungi, is currently unknown.

The goals of this study were to: 1) characterize the mating types of *V. dahliae* from a large collection of phytopathogenic isolates; 2) determine whether isolates of opposite mating types are present concurrently in the same habitat; 3) determine whether genetically identical multilocus microsatellite types contain both *MAT* idiomorphs; 3) determine if the complete genome sequence of *V. dahliae* strain Ls 17 contains orthologs of fungal sex-related genes; 4) test whether such genes are constitutively expressed in both mating types under laboratory conditions; and 5) estimate the extent of positive (relaxed) and purifying selection in a subset of sex-related genes in *V. dahliae*, relative to fungi with known sexual stages.

## Results and Discussion

### Molecular assays to identify *Verticillium* species, *MAT* type, and multilocus microsatellite types

All isolates used in this study were identified as the phylogenetic species *V. dahliae sensu stricto* [35]. The frequency of *MAT* idiomorphs was extremely skewed towards an overabundance of *MAT1-2* (Table S1). The *MAT1-1* idiomorph was only observed in 1% (12/1120) of isolates characterized. The *MAT1-1* isolates comprised eight isolates from commercial spinach seed lots from Washington State, USA, two isolates from a commercial artichoke field in California, and one isolate each from two commercial tomato field in CA (Table S1).

Complete multilocus microsatellite types were generated for 941 isolates; all 12 *MAT1-1* isolates had different MLMTs, whereas 410 different MLMTs were observed for *MAT1-2* isolates. Thus, after clone correction, 97% (410/422) isolates were *MAT1-2*. Nine of the *MAT1-1* MLMTs were identical to MLMTs of one or more *MAT1-2* isolates (Table 1). Of the nine MLMTs that comprised both mating types, three of them were found to have overlapping ecological niches. That is, they were collected at the same time from the same location and were isolated from the same substrate (artichoke, spinach seed, and tomato) (Table S1). The presence of multilocus genotypes common to both mating types has been interpreted as evidence of sexual recombination [11], [47]. However, this interpretation assumes no homoplasy, and assumes that isolates of opposite mating types did not acquire the same alleles at the thirteen loci independently through mutation.

**Table 1:** Ecological characteristics of multilocus microsatellite types that comprised isolates of both mating types.

MLMT	Alleles for 13-locus MLMT a	*MAT1-1* (n)^ b^	*MAT1-2* (n)^c^	*MAT1-1* plant hosts	*MAT1-2* plant hosts	*MAT1-1* origins	*MAT1-2* origins
1	366.315.369.333.329.577.361.350.367.373.392.334.317	1	2	Tomato	Lettuce	CA, USA	CA, USA
2	372.299.369.301.263.521.333.330.367.289.332.246.277	1	1	Spinach seed	Spinach seed	WA, USA	Netherlands
3	372.303.369.301.263.521.333.330.367.283.332.246.277	1	2	Spinach seed	Spinach seed	WA, USA	WA, USA
4	372.303.369.301.263.521.333.330.387.295.332.246.277	1	5	Spinach seed	Olive, Spinach seed	WA, USA	Denmark, Italy, WA, USA
5	378.299.369.301.263.521.333.330.387.283.332.246.277	1	45	Tomato	Cotton, Spinach seed, Tomato	CA, USA	Chile, CA and WA USA
6	378.299.369.301.263.521.333.330.387.301.332.246.277	1	18	Spinach seed	Spinach seed	WA, USA	WA, USA
7	378.315.376.301.263.513.361.330.367.301.332.246.277	1	1	Spinach seed	Spinach seed	WA, USA	WA, USA
8	384.299.369.301.263.521.333.330.367.289.332.246.277	1	12	Spinach seed	Spinach seed	WA, USA	WA, USA
9	384.299.376.301.263.545.333.330.367.295.401.250.277	1	11	Artichoke	Artichoke, Lettuce	CA, USA	CA, USA

a “0” indicates no amplification at locus; alleles are presented in the order: VD2.VD1.VD9.VD11.VD92.VD97.VD69.VD12.VD27.VD73.VD8.VD10.VD3. ^b, c^ Total number of *MAT1-1*, *MAT1-2* isolates for each microsatellite type.

### *Verticillium* genome queries and ortholog searches

Out of 93 sex-related genes considered, 88 were found in the *V*. *dahliae* genome (Table 2). The five genes not found in *V. dahliae* genome searches were the *N. crassa* accessions NCU09793, NCU04329, which are DNA helicase and repair proteins, respectively, and *S. cerivisae* accessions YIL072W, YGL033W, and YGL183C, which correspond to *HOP1*, *HOP2* and *MND1*. Since no orthologs to *HOP1*, *HOP2* or *MND1* were found among any of the Sordariomycetes in the FUNGIPath database, including the sexual fungi *Neurospora crassa, Podospora anserina,* and *Nectria haematococca*, it is reasonable to speculate that these three genes are not required for a fully functional sexual cycle for taxa in this group.

**Table 2:** *Verticillium dahliae* orthologs of *Neurospora crassa, Saccharomyces cerevisiae, Podospora anserina* sex-related genes.

Gene annotation/putative function	*V. dahliae* *accession*	Synonym	Otheraccession	Annotatedfungal species
**Meiosis**				
**Double-strand DNA breaks formation and processing**				
Meiotic recombination protein REC12	VDAG_09359	SPO11	NCU01120	*Neurospora crassa*
Meiotic recombination protein REC4	VDAG_07486	SKI8	NCU03517	*Neurospora crassa*
DEAD/DEAH box DNA helicase MER3	NA		NCU09793	*Neurospora crassa*
Splicing factor 3B subunit 4	VDAG_08454		NCU04182	*Neurospora crassa*
Double-strand break repair protein MUS23	VDAG_07631		NCU08730	*Neurospora crassa*
DNA repair protein RAD50	VDAG_06865	USV6	NCU00901	*Neurospora crassa*
DNA repair protein of the MRE11 complex	NA		NCU04329	*Neurospora crassa*
**Single strand invasion**				
DNA repair protein RAD51	VDAG_08796	MEI3	NCU02741	*Neurospora crassa*
DNA repair and recombination protein RAD52	VDAG_00265	MUS11	NCU04275	*Neurospora crassa*
DNA repair and recombination protein RAD54	VDAG_02310		NCU11255	*Neurospora crassa*
Replication factor-A protein1	VDAG_08650	RPA1	NCU03606	*Neurospora crassa*
Replication factor-A protein 2	VDAG_10269		NCU07717	*Neurospora crassa*
Strand exchange protein RAD55p	VDAG_00585		NCU08806	*Neurospora crassa*
DNA-repair protein XRCC3	VDAG_07164		NCU01771	*Neurospora crassa*
**DNA damage checkpoint**				
Genome integrity checkpoint protein	VDAG_05896		NCU00274	*Neurospora crassa*
Cell cycle checkpoint protein RAD17	VDAG_03081		NCU00517	*Neurospora crassa*
**Proteins involved in crossing over**				
DNA mismatch repair protein	VDAG_07693		NCU05385	*Neurospora crassa*
DNA mismatch repair protein MUTS	VDAG_02856	MSH4	NCU10895	*Neurospora crassa*
DNA mismatch repair MUTS family	VDAG_08845	MSH5	NCU09384	*Neurospora crassa*
ATP-dependent helicase SGS1	VDAG_04304	MUS19	NCU08598	*Neurospora crassa*
Meiosis specific protein	VDAG_05193		NCU10836	*Neurospora crassa*
DNA repair protein RAD16	VDAG_01793	MUS38	NCU07440	*Neurospora crassa*
DNA repair protein RAD13	VDAG_00986		NCU07498	*Neurospora crassa*
**Synaptonemal complex**				
Histone H2A.Z	VDAG_07626		NCU05347	*Neurospora crassa*
Structural maintenance of chromosome: SMC protein	VDAG_01776		NCU09065	*Neurospora crassa*
Structural maintenance of chromosome: SMC protein	VDAG_09439		NCU02402	*Neurospora crassa*
Exodeoxyribonuclease	VDAG_02157		NCU06089	*Neurospora crassa*
Casein kinase I	VDAG_02638		NCU00685	*Neurospora crassa*
Nucleotide excision repair protein RAD23	VDAG_09770	RAD23	NCU07542	*Neurospora crassa*
ATP-dependent DNA helicase SRS2	VDAG_01559	MUS50	NCU04733	*Neurospora crassa*
**Mismatch repair proteins**				
DNA mismatch repair protein MSH2	VDAG_02253	MSH2	NCU02230	*Neurospora crassa*
DNA mismatch repair protein MSH3	VDAG_04229	MSH3	NCU08115	*Neurospora crassa*
DNA mismatch repair protein MSH6	VDAG_01192	MSH6	NCU08135	*Neurospora crassa*
DNA mismatch repair protein PMS1	VDAG_09041		NCU08020	*Neurospora crassa*
DNA mismatch repair protein MUTL	VDAG_08805		NCU09373	*Neurospora crassa*
**Resolution of recombination intermediates**				
Protein involved in DNA repair and recombination	VDAG_05488		NCU04047	*Neurospora crassa*
Crossover junction endonuclease MUS81	VDAG_03195	MUS81	NCU07457	*Neurospora crassa*
GIY-YIG catalytic domain containing protein	VDAG_09308		NCU01236	*Neurospora crassa*
DNA topoisomerase	VDAG_04479		NCU09118	*Neurospora crassa*
DNA topoisomerase	VDAG_00604		NCU06338	*Neurospora crassa*
DNA topoisomerase	VDAG_06518		NCU00081	*Neurospora crassa*
**Non-homologous end joining**				
Ku70 protein	VDAG_10247	MUS51	NCU08290	*Neurospora crassa*
Ku80 protein	VDAG_06524	MUS52	NCU00077	*Neurospora crassa*
**Other**				
Protein required for meiotic recombination	VDAG_07839		NCU04415	*Neurospora crassa*
Repeat-induced point mutation gene	VDAG_05093	RID	NCU02034	*Neurospora crassa*
Synaptonemal complex protein HOP1	NA		YIL072W	*Saccharomyces cerevisiae*
Interhomolog meiotic recombination HOP2	NA		YGL033W	*Saccharomyces cerevisiae*
Interhomolog meiotic recombination MND1	NA		YGL183C	*Saccharomyces cerevisiae*
**Cohesion**				
**Adherin**				
Subunit of cohesin loading factor	VDAG_00695		NCU05250	*Neurospora crassa*
**Chromosome cohesion**				
Cohesin complex subunit	VDAG_04575		NCU01323	*Neurospora crassa*
Chromosome segregation protein SUDA	VDAG_06558		NCU07554	*Neurospora crassa*
Cohesin complex subunit required for sister chromatid cohesion	VDAG_08327		NCU01247	*Neurospora crassa*
Double-strand-break repair protein RAD21	VDAG_08702	RAD21	NCU03291	*Neurospora crassa*
Rec8 protein	VDAG_02664	REC8	NCU03190	*Neurospora crassa*
Protein required for establishment andmaintenance of sister chromatid cohesion	VDAG_03579	V-SNARE	NCU00242	*Neurospora crassa*
**Separin**				
Separin	VDAG_05810		NCU00205	*Neurospora crassa*
**Condensins**				
Nuclear condensin complex subunit Smc2	VDAG_00648		NCU07679	*Neurospora crassa*
Nuclear condensin complex subunit Smc4	VDAG_10489		NCU09063	*Neurospora crassa*
Condensin	VDAG_09545		NCU09297	*Neurospora crassa*
Condensin subunit Cnd3	VDAG_06322		NCU06216	*Neurospora crassa*
**Chromosome segregation**				
Spindle pole body component alp14	VDAG_10219		NCU04535	*Neurospora crassa*
HEC/Ndc80p family protein	VDAG_10087		NCU03899	*Neurospora crassa*
Chromosome segregation protein	VDAG_09035		NCU07984	*Neurospora crassa*
Swi3 domain-containing protein	VDAG_04932		NCU01858	*Neurospora crassa*
Carboxy-terminal kinesin 2	VDAG_09024		NCU04581	*Neurospora crassa*
Tubulin alpha chain	VDAG_04060		NCU09132	*Neurospora crassa*
Tubulin gamma chain	VDAG_01827	TBG	NCU03954	*Neurospora crassa*
Tubulin alpha chain	VDAG_04060	TBA2	NCU09468	*Neurospora crassa*
**Anaphase-promoting complex**				
Anaphase-promoting complex/cyclosome subunit APC1	VDAG_09956		NCU05901	*Neurospora crassa*
Anaphase-promoting complex protein	VDAG_02447		NCU01963	*Neurospora crassa*
Anaphase-promoting complex subunit CUT9	VDAG_01327		NCU01377	*Neurospora crassa*
WD repeat-containing protein slp1	VDAG_06090		NCU02616	*Neurospora crassa*
Anaphase-promoting complex subunit 8	VDAG_08529		NCU01174	*Neurospora crassa*
Nuclear protein BIMA	VDAG_05870		NCU00213	*Neurospora crassa*
Anaphase-promoting complex subunit 10	VDAG_07093		NCU08731	*Neurospora crassa*
WD repeat-containing protein SRW1	VDAG_04583		NCU01269	*Neurospora crassa*
Meiosis-specific APC/C activator protein AMA1	VDAG_01235		NCU01572	*Neurospora crassa*
**Transcription factor and gene regulation**				
Meiosis-specific transcription factor	VDAG_00592		NCU09915	*Neurospora crassa*
Histone-lysine N-methyltransferase	VDAG_10394		NCU06266	*Neurospora crassa*
Ankyrin repeat protein	VDAG_06433		NCU00388	*Neurospora crassa*
SNF2 family ATP-dependent chromatin-remodeling factor SNF21	VDAG_06547		NCU06488	*Neurospora crassa*
**Signal transduction**				
Calcium/Calmodulin-dependent protein kinase	VDAG_04474		NCU09123	*Neurospora crassa*
Protein kinase GSK3	VDAG_08431		NCU04185	*Neurospora crassa*
Serine/Threonine-protein kinase RIM15	VDAG_03223		NCU07378	*Neurospora crassa*
**Pheromone proteins essential for mating**				
Pheromone processing	VDAG_05762	STE23	YLR389C	*Saccharomyces cerevisiae*
Peptide pheromone maturation	VDAG_06292	RCE1	YMR274C	*Saccharomyces cerevisiae*
Pheromone processing	VDAG_09962	AFC1	YJR117W	*Saccharomyces cerevisiae*
Protein processing	VDAG_00116	KEX1	YGL203C	*Saccharomyces cerevisiae*
Pheromone receptor	VDAG_05622	PRE2	Pa_4_1380	*Podospora anserina*
Farnesyltransferase subunit beta	VDAG_05598	RAM1	Pa_4_7760	*Podospora anserina*
Putative ABC transporter expressed in the mitochondrial inner membrane	VDAG_01200	STE6	Pa_5_11640	*Podospora anserina*

### SELECTON analyses of positive and purifying selection in sex-related genes of *V. dahliae*

**Figure 1:**
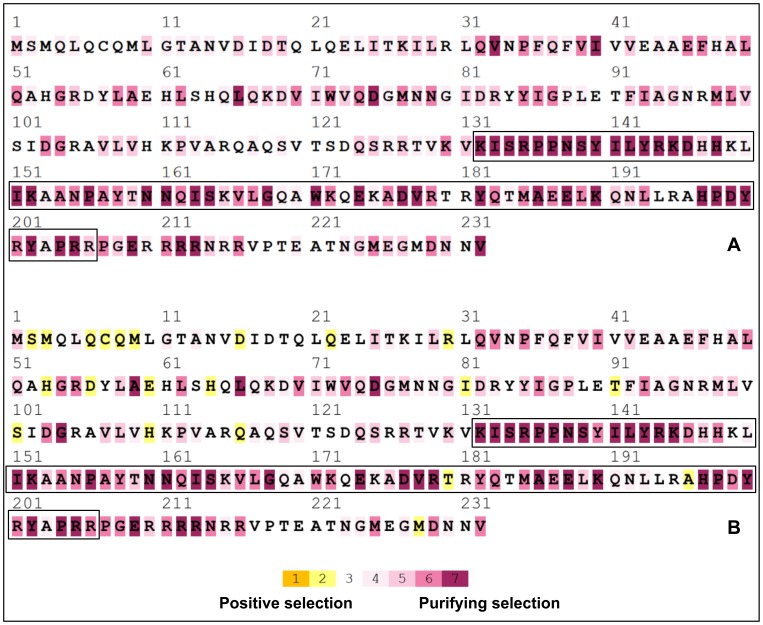
Color-coded results of SELECTON analyses of *Verticillium dahliae MAT1-1-1*, compared to sequences from nine different sexual fungi in the *Pezizomycotina*. Shades of yellow (colors 1 and 2) indicate a Ka/Ks ratio>1 (positive selection), and shades of purple (colors 3 through 7) indicate a Ka/Ks ratio<1 (purifying selection); A) results from the M8 model; B) results of the MEC model; amino acid sequence of the α domain is indicated by black border.

**Figure 2:**
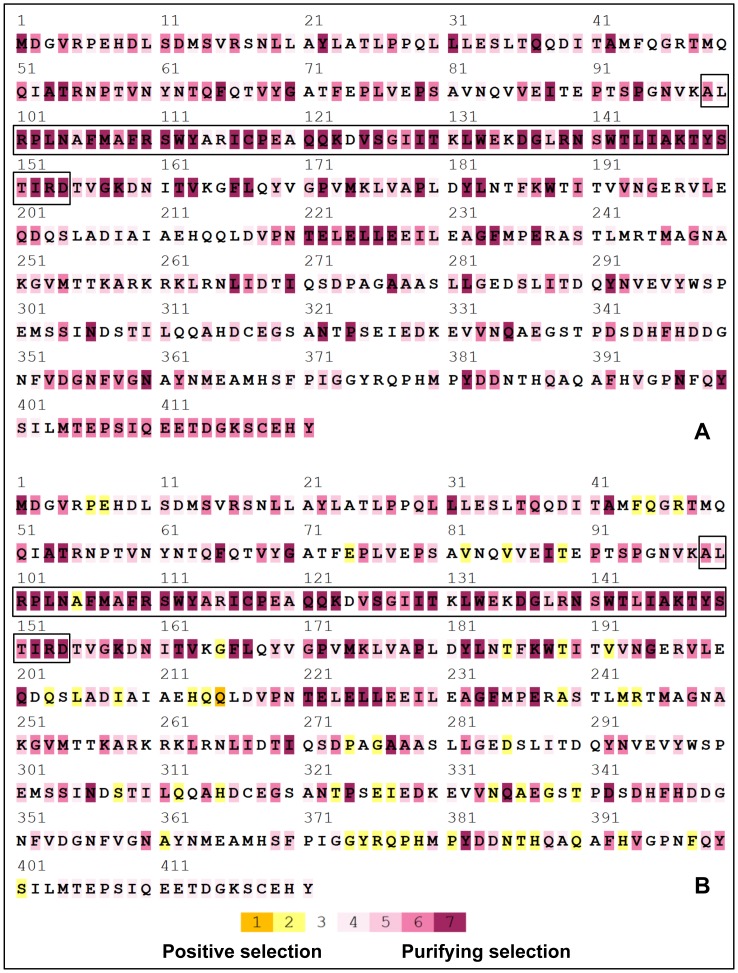
Color-coded results of SELECTON analyses of *Verticillium dahliae MAT1-2-1*, compared to sequences from nine different sexual fungi in the *Pezizomycotina*. Shades of yellow (colors 1 and 2) indicate a Ka/Ks ratio>1 (positive selection) and shades of purple (colors 3 through 7) indicate a Ka/Ks ratio<1 (purifying selection); A) results from the M8 model; B) results of the MEC model; amino acid sequence of the HMG domain is indicated by black border.

Selective pressures were estimated in 20 *V*. *dahliae* genes, including *MAT1-1-1* and *MAT1-2-1*. The subset of 20 genes chosen for SELECTON analysis were distributed in the *V. dahliae* genome on chromosomes 1, 2, 3, 4, 5, 7 and 8. No codons under positive selection were detected in either *MAT1-1-1* or *MAT1-2-1* or any of the other 18 genes using the M8 model (Figure 1A, Figure 2A). However, using the MEC model, positive selection was detected in 12/20 genes investigated (Figure 1B, Figure 2B, Figure S1). Likelihood ratio tests between the MEC and M8a models revealed that in all cases, the AIC score of the MEC model was lower than the M8a model.

Using the MEC model, *Verticillium dahliae MAT1-1-1* contained 12% of codons under positive selection and 34% of codons under strong purifying selection (Table 3); *V. dahliae MAT1-2-1* contained 9% of codons under positive selection and 35% of codons under strong purifying selection (Table 4). Of the 21 codons under positive selection in *V*. *dahliae MAT1-1-1*, only 3 were within the highly conserved α domain (Figure 1B); similarly of the 51 codons under positive selection in *V. dahliae MAT1-2-1*, only 1 was within the highly conserved HMG domain (Figure 2B). When only sequences from sexual fungi were considered, *MAT1-1-1* codons under positive and purifying selection ranged from 9–15% and 33–43% respectively (Table 3), whereas *MAT1-2-1* codons under positive and purifying selection ranged from 12–22% and 21–30% respectively (Table 4). Thus, the extent and type of selection estimated for *V. dahliae MAT* genes were comparable to the estimates for *MAT* genes from sexual fungi. Interestingly, *MAT1-1-1* from the putatively asexual *P. fulva* contained the highest relative numbers of codons under positive selection and the lowest under strong purifying selection (Table 3); however, *P. fulva MAT1-2-1* Ka/Ks estimates were similar to sexual fungi (Table 4).

**Table 3:** Comparison of codons under positive (relaxed) and purifying selection in *MAT1-1-1*, in a variety of fungi in the subphylum *Pezizomycotina* using the MEC model.

Fungal taxon				
	*MAT1-1 −1* accession no.	Transcript length(codons)	Codons under positiveselection	Codons under strong purifyingselection
*Verticillium dahliae* 1	NCBI GenBank AB505215	421	51 (12%)	146 (34%)
*Aspergillus fumigatus*	NCBI GenBank AY898660	369	45 (12%)	125 (33%)
*Aspergillus nidulans* 2	ANID_02755	362	45 (12%)	129 (35%)
*Cochliobolus heterostrophus*	NCBI GenBank X68399	384	46 (12%)	129 (33%)
*Eupenicillium crustaceum* 2	NCBI GenBank FR729897	343	34 (9%)	121 (35%)
*Fusarium graminearum* 2	FGSG_08892	345	53 (15%)	126 (36%)
*Fusarium verticillioides*	FVEG_02491	383	54 (14%)	129 (33%)
*Histoplasma capsulatum*	HCAG_09679	305	34 (11%)	107 (35%)
*Nectria heamatococca*	NCH17696	214	20 (9%)	92 (43%)
*Penicillium chrysogenum*	PC_255945071	342	34 (9%)	119 (34%)
*Sclerotinia sclerotiorum*	SS1G_04004	258	35 (13%)	91 (35%)
*Passalora fulva* 3	DQ659350	358	60 (16%)	98 (27%)

1 SELECTON results for the putatively asexual fungus *V. dahliae* were calculated by analyzing a *MAT1-1-1* codon sequence alignment including sequences from all other fungi listed except *P. fulva*. Results for the ten species *A. fumigatus – S. sclerotiorum* were calculated using a codon alignment of only these ten species.

2 Homothallic fungus.

3 Results for the putatively asexual fungus *P. fulva* were calculated by analyzing a *MAT1-2-1* codon sequence alignment including sequences from all other fungi listed except *V. dahliae*.

**Table 4:** Comparison of codons under positive (relaxed) and purifying selection in *MAT1-2-1*, in a variety of fungi in the subphylum *Pezizomycotina* using the MEC model.

Fungal taxon	*MAT1-2-1* accession no.	Transcriptlength (codons)	Codons underpositive selection	Codons under strongpurifying selection
*Verticillium dahliae* 1	VDAG_02444	232	21 (9%)	81 (35%)
*Chaetomium globosum*	CHGG_03580	342	74 (22%)	101 (30%)
*Aspergillus nidulans* 2	ANID_04734	318	70 (22%)	95 (30%)
*Colletotrichum graminicola*	GLRG_04643	238	42 (18%)	76 (32%)
*Fusarium graminearum* 2	FGSG_08893	253	52 (21%)	76 (30%)
*Fusarium sacchari*	NCBI GenBank JF776855	227	48 (21%)	69 (30%)
*Magnaporthe grisea*	MG_02978	437	52 (12%)	150 (34%)
*Ophiostoma novo-ulmi*	NCBI GenBank FJ959052	183	33 (18%)	59 (32%)
*Podospora anserina*	Pa_1_20590	582	74 (13%)	124 (21%)
*Penicillium chrysogenum*	NCBI GenBank AM904545	303	64 (21%)	91 (30%)
*Trichoderma ressei*	TRI14830	241	46 (19%)	56 (23%)
*Passalora fulva* 3	DQ659351	384	45 (11%)	133 (34%)

1 SELECTON results for the putatively asexual fungus *V. dahliae* were calculated by analyzing a *MAT1-2-1* codon sequence alignment including sequences from all other fungi listed except *P. fulva*. Results for the ten species *C. globosum – T. reseei* were calculated using a codon alignment of only these ten species.

2 Homothallic fungus.

3 Results for the putatively asexual fungus *P. fulva* were calculated by analyzing a *MAT1-2-1* codon sequence alignment including sequences from all other fungi listed except *V. dahliae*.

In addition to the *MAT* genes, Ka/Ks patterns were investigated in 18 other sex-related genes (Table 5, Table S2). The percentage of codons in *V. dahliae* genes under positive and strong purifying selection ranged from 0–5% and 35–62%, respectively. Six genes, *KEX1*, *MEI3*, *RAD21*, *RAD54*, *STE23*, and *V-SNARE* contained no codons under positive selection using either the M8 or MEC model (Table 5, Figure S1).

**Table 5:** Comparison of codons under positive (relaxed) and purifying selection in 18 sex-related genes in *Verticillium dahliae* using the MEC model.

V. dahliae accession^1^	Locus	Transcript length (codons)	Codons under positive selection	Codons under strong purifying selection
VDAG_00116	*KEX1*	384	0 (0%)	154 (40%)
VDAG_08796	*MEI3*	354	0 (0%)	142 (40%)
VDAG_02856	*MSH4*	843	40 (3%)	313 (37%)
VDAG_08845	*MSH5*	863	8 (1%)	340 (39%)
VDAG_01559	*MUS50*	1166	5 (<1%)	462 (40%)
VDAG_01559	*MUTL*	704	2 (<1%)	281 (40%)
VDAG_08702	*RAD21*	530	0 (0%)	258 (49%)
VDAG_02310	*RAD54*	651	0 (0%)	261 (40%)
VDAG_05598	*RAM1*	469	4 (<1%)	185 (39%)
VDAG_06292	*RCE1*	304	1 (<1%)	122 (40%)
VDAG_02664	*REC8*	452	33 (2%)	281 (62%)
VDAG_01783	*RID*	957	66 (5%)	343 (36%)
VDAG_07486	*SKI8*	336	1 (<1%)	135 (40%)
VDAG_09359	*SPO11*	425	38 (3%)	149 (35%)
VDAG_05762	*STE23*	941	0 (0%)	377 (40%)
VDAG_06443	*STE24*	300	21 (1%)	107 (36%)
VDAG_01200	*STE6*	1416	23 (1%)	526 (37%)
VDAG_03579	*V-SNARE*	128	0 (0%)	51 (40%)

1 Fungal taxa and gene accessions used to estimate selective pressures in *V. dahliae* genes are provided in Table S2. Color-coded SELECTON results for each gene are provided in Figure S1.

### Expression of sex-related genes based on RT-PCR

**Figure 3:**
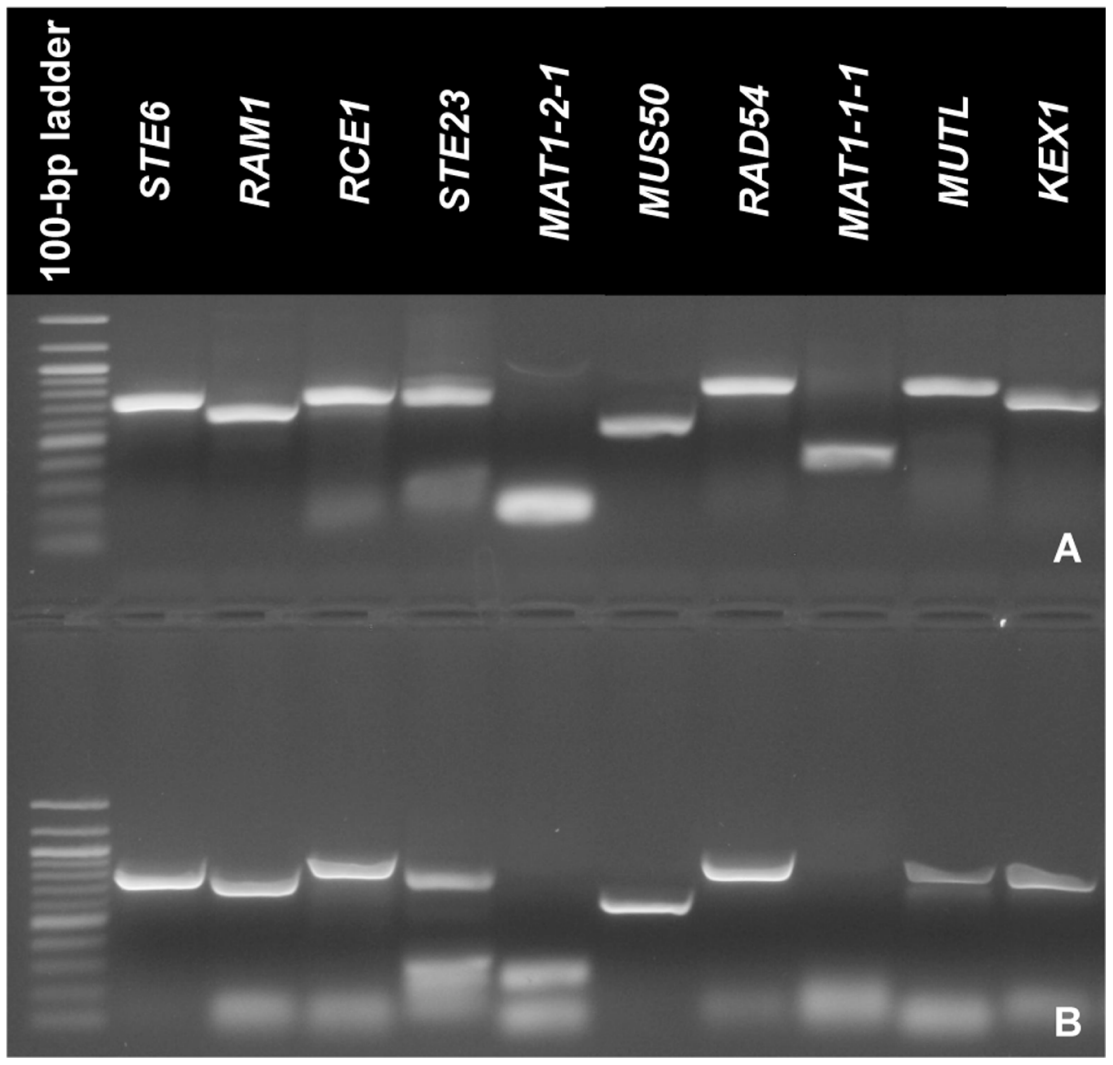
Reverse-transcriptase PCR results of 10 *Verticillium dahliae* orthologs of genes associated with the sexual cycle in model fungal systems; gene names are provided for each lane; A) RT-PCR results from *V. dahliae* strain 58 (*MAT1-1)*. B) RT-PCR results from *V. dahliae* strain Ls 17 (*MAT1-2*);

RT-PCR using RNA from both mating types of *V. dahliae* successfully amplified all 10 sex-related genes investigated (Figure 3). As expected, RNA from *MAT1-1-1* and *MAT1-2-1* only amplified from the strain that carried the respective *MAT1-1* and *MAT1-2* idiomorph (Figure 3). DNAse was used to treat extracted RNA, and no amplification was observed in reactions with reverse transcriptase omitted, indicating that DNA contamination was not present in the reactions (gels not shown). Since fungal isolates were cultured independently, it appears that *V*. *dahliae* expressed these genes during vegetative growth on PDA in the absence of a compatible culture of opposite mating type.

### Conclusions

The overabundance of *MAT1-2* in *V*. *dahliae* has been reported on multiple scales, from heavily sampled single agricultural fields to larger scales such as countries. This phenomenon may be partly explained by clonal expansion of certain successful, highly fit genotypes which do not require sexual reproduction to complete the disease cycle [23], [31], unlike some other plant pathogens. Nevertheless, in two field sites in coastal California and in commercial spinach seed lots from WA, identical multilocus microsatellite types comprising both mating types were found, indicating at the very least, that both *MAT1-1* and *MAT1-2* co-occur in some niches currently.

The sample of *V. dahliae* characterized in the current study was biased toward virulent, phytopathogenic isolates collected from diseased plant tissue, because most were isolated from plants with visible wilt symptoms in agricultural settings. This raises the hypothesis that *V. dahliae MAT1-2* may be associated with higher virulence on some, if not all hosts, which is a phenomenon that has been reported in other fungal systems [48]–[51]. Preliminary data on the virulence of isolates from both idiomorphs originally collected from tomato suggest that *MAT1-2* isolates are significantly more virulent than *MAT1-1* isolates (Subbarao, unpublished data). A more comprehensive analysis of the virulence of the two idiomorphs is required to confirm these results, however with more experiments and by investigating the mating-type structure in populations of non-pathogenic, endophytic *V. dahliae* [52], [53].

Although the current study clearly documents patterns of purifying selective pressures in protein coding regions of the sex-related *V. dahliae* genes investigated, it is possible that there are mutations in non-coding, regulatory regions of the genome that affect the level, timing or location of sex-related gene expression and therefore hinder the sexual cycle. Furthermore, it is possible that genes that were originally associated with sexual reproduction in ancestral populations have evolved new functions, and this is the reason they are being maintained under selection. Yet, it has been previously supposed that the presence of the majority, if not all, of the meiosis-specific genes in the genome of a microorganism is the “strongest indicator” that genes are maintained for meiosis and sex, even if it is rare [10]. The *V. dahliae* genome is clearly replete with orthologs to genes known for their roles in pathways associated with the sexual cycle. Further, the SELECTON analyses provide evidence that sex-related genes are not in the process of becoming pseudogenes.

The production of actual sexual structures *in vitro* currently remains a mystery in *V. dahliae*, possibly due to the lack of research into the growth medium content requirements, such as nutrient (i.e. carbon) content and pH, which are highly variable for sexual fungi in the *Pezizomycotina* [5], [16]. Nevertheless, the genomic evidence presented in the current study, taken together with previous studies of population structure and recombination [23], is compelling and could be reasonably interpreted as evidence of an ancestral or rare sexual cycle in this predominantly asexual species.

## Materials and Methods

### Fungal culture maintenance and DNA extraction for *MAT* characterization

In this study, 1120 isolates of *V. dahliae*, collected from 10 different countries, were characterized for mating type (Table S1). No specific permissions were required for isolating *Verticillium* from any of the regions in the current study. The field isolations did not involve endangered or protected species. Importation of *Verticillium* cultures was performed under the appropriate USDA-APHIS permits (P526P-11-02218, P526P-11-02476, P526P-11-02806). *Verticillium* cultures were originally cultivated on semi–selective NP–10 medium [54], and then single-conidium purified and transferred to potato dextrose agar (PDA). Cultures were stored long-term as spore suspensions in 25% glycerol at −20°C. Mycelia for DNA extraction were grown in 250 ml Erlenmeyer flasks containing 50 ml potato dextrose broth (PDB). Each flask was inoculated with a piece of PDA culture with an approximate surface area of 1 cm^2^. Mycelia from PDB were harvested after 10 days, washed with sterile distilled water, dried using paper towels, lyophilized, and ground to a fine powder using a high-speed mixer mill (Model MM301; Retsch Inc., Newtown, PA). Genomic DNA of each isolate was extracted using a FastDNA Kit (MP Biomedicals LLC, Solon, OH) following the manufacturer’s instructions. A Nano Drop (Model ND–1000, Thermo Scientific Inc., Waltham, MA) was used to quantify DNA extractions, which were diluted to 10 ng/µl, and stored in a freezer at –20°C until needed for PCR assays.

### Molecular assays to identify *Verticillium* species and mating type

All isolates used in this study were identified as *V*. *dahliae* using a *Verticillium* species-specific multiplex as previously described [35]. Mating types were determined for 1120 *V. dahliae* isolates PCR assay with the previously developed primers Alf3 (CGATCGCGATATCGGCAAGG), MAT11r (CAGTCAGATCCAACCTGCTGGCC), HMG21f (CGGCCGCCCAATTCGTACATCC) and MAT21r (CATGCCTTCCATGCCATTAGTAGCC). These primers amplify a ∼600-bp fragment from *MAT1*–*1*–*1* isolates and a ∼300-bp fragment from *MAT1*–*2*–*1* isolates, as previously described [29], [35], [37]. PCR assays to characterize mating types were performed in 25 µl reactions using GoTaq Green Mastermix (Promega, Madison, WI). All PCR assays in this study were performed in a PTC-100 Peltier Thermal cycler (MJ Research, Inc., Waterman, MA). For mating type multiplex PCR, the following thermal profile was used: 2 min initial denaturation at 94°C, 35 cycles of 10 sec at 94°C, 20 sec at 57°C, and 1 min at 72°C, followed by a final extension of 7 min at 72°C. PCR amplicons were stained with 5 µl SyberGold (Invitrogen Life Technologies, Carlsbad, CA), and aliquots were loaded in a 1.5% (wt/vol) agarose gel and run for 120 min at 75 V in 0.5% TBE buffer [55]. A 100–bp DNA ladder (Invitrogen Life Technologies, Carlsbad, CA) was included in each gel and a transilluminator (Ultra-Violet Products, Ltd., Upland, CA) was used to visualize PCR products.

### Multilocus microsatellite genotyping

Thirteen previously developed microsatellite loci were used in this study: VD1, VD2, VD3, VD8, VD9, VD10, VD11, VD12, VD27, VD69, VD73, VD92 and VD97 [56] which were developed using the *V. dahliae* strain Ls 17 complete genome sequence [30], [37]. For all microsatellite loci, PCR was performed in 20 µl total volumes containing 4 µl of sterile, distilled water, 2 µl of 10 ng/µl genomic DNA, 2 µl each of 10 µM reverse and forward primer, and 12.5 µl of GoTaq Green PCR mix (Promega Inc., Madison, WI). Published thermocycling parameters were used as previously described [30]. PCR amplicons labeled with up to four fluorophores FAM, HEX, ROX and TAMRA (Invitrogen, Carlsbad, CA) were pooled [57]. One µl of the pooled amplicons was then combined with Hi-Di formamide and 0.3 µl of LIZ–500 size standard and separated on an ABI 3100 capillary electrophoresis genetic analyzer (Applied Biosystems, Carlsbad, CA) at the University of California-Davis DNA Sequencing Facility, Davis CA. The peaks in were scored using the GeneMarker software (SoftGenetics, State College, PA).

To assess reliability of microsatellite allele calls using capillary electrophoresis [58], 192 microsatellite amplicons representative of all 13 loci were arbitrarily selected for DNA sequencing using unlabeled forward and reverse primers. Amplicons from *V. dahliae* strain Ls 17 were also generated and compared to the results reported from the same strain in previous studies [30], [56]. Different amplicon sizes at each locus were considered unique. Alleles were compiled across loci into multilocus microsatellite types (MLMTs).

### *Verticillium* genome queries and ortholog searches

The FUNGIpath ortholog database was queried using a panel of 93 genes that have been characterized for functions related to sexual reproduction in the fungal model systems *Neurospora crassa, Saccharomyces cerevisiae,* and *Podospora anserina*. The set of 93 genes comprised the two mating type genes *MAT1-1-1* and *MAT1-2-1*, 81 previously described *Neurospora crassa* genes associated with meiosis [36], [59], [60] which were retrieved from the *Neurospora* Genome Database [61], [62], four previously described *Saccharomyces cerevisiae* pheromone-related genes *STE23*, *RCE1*, *AFC1*, *KEX1* [63], which were retrieved from the *Saccharomyces* Genome Database [24], [64], and three *Podospora anserina* pheromone-related genes *PRE2*, *RAM1*, *STE6* [63] which were retrieved from the *Podospora anserina* Genome Database [25], [65]. Since *V. dahliae* is heterothallic and the sequenced strain contains only *MAT1-2-1*, a sequence of *V. dahliae MAT1-1-1* was obtained through National Center of Bioinformatics (NCBI) GenBank, Accession AB505215 [27]. Finally, three additional *Saccharomyces cerevisiae* genes broadly associated with meiosis in eukaryotes (*HOP1*, *HOP2*, and *MND1*) [10] were queried against the FUNGIpath database. For FUNGIpath ortholog database searches, either gene accession ids. or amino acid sequences were used as input [41]. In this way, *V. dahliae* genes were verified as orthologous to genes from sexual fungi. Ortholog gene accession ids. from other fungi in the *Pezizomycotina* were noted and downloaded from the respective genome databases for subsequent analyses.

### Primer design

After identifying orthologs to sex-related genes in the genome of *V*. *dahliae*, coding sequences of *MAT* genes and eight other genes associated with meiosis in other systems were arbitrarily chosen and downloaded from the Broad Institute website [41]. Forward and reverse primers were designed to amplify ∼500 to 1000-bp targets within coding sequences for 8 of the genes, whereas the previously described primers Alf3-MAT11r and HMG21f-MAT21r [29] were used to amplify *MAT1-1-1* and *MAT1-2-1*, respectively (Table 6).

**Table 6:** Primers used to amplify *V. dahliae* sex-related genes with RT-PCR.

Gene name	*V. dahliae* accession^1^	Fw primer 5′–3′	Rv primer 5′–3′
*MAT1-1-1*	NA 1	CGATCGCGATATCGGCAAGG	CAGTCAGATCCAACCTGCTGGCC
*MAT1-2-1*	VDAG_02444	GCAATGTCAGATGCTCGGTA	CTGCGAGATAATCACGACCA
*STE6*	VDAG_01200	GCAAACTTCTCACCCTCTGC	CAGGTCGTCTCCCACTTTGT
*MUS50*	VDAG_01559	CGACCTTATCGGCGATCTAC	CTCTCTTCTGGGTCGACAGG
*RAD54*	VDAG_02310	GCAAACGAGCTTGTCAAGTG	GGTTGCAGAGCTTCTTGAGG
*RAM1*	VDAG_05598	GCTTCTACGCCAGCAGACAC	GTCGACTTCACCGCCATAC
*STE23*	VDAG_05762	ACAGGTTCTCGTCACCATCC	GGACATGGTGTCAATGATCG
*RCE1*	VDAG_06292	ACAGAGGAGCTGCTTTTTCG	TCCACCACGCTTCTTGAACT
*MUTL*	VDAG_08805	AAGGCTCTACCGCCAATTTT	TCATCGTTTCGTCTGCTCTG
*MSH5*	VDAG_08845	CGGGACATTTACCGATGAAC	TCCTCAGCATCCCTCAGTCT

1 The genome of *V. dahliae* strain Ls 17 contains only *MAT1-2-1. MAT1-1-1* sequence obtained from NCBI GenBank.

### RNA Extraction and RT-PCR

The two *V. dahliae* isolates 58 (*MAT1-1-1*) and Ls 17 (*MAT1-2-1*) were grown on PDA. For each culture, after ten days, 3 ml of sterile distilled water was poured onto the culture surface and spread with a plate spreader. One ml of the resulting conidia and hyphal suspensions was transferred to a 47 mm nitrocellulose membrane (0.45 µm pore size; Whatman, Maidstone, England) overlaid on a PDA plate. Cultures were maintained in the dark at 25°C. After 10 days, the nitrocellulose membranes covered in fungal tissue were harvested with sterilized forceps and ground to a fine powder in liquid nitrogen using a mortar and pestle. Total RNA was extracted from 100 mg of the ground powder using TRIzol Reagent (Life Technologies, Carlsbad, CA) following the manufacturer’s protocol. Total RNA extracts were treated with TURBO DNase (Life Technologies, Carlsbad, CA) following the manufacturer’s protocol, in order to degrade genomic DNA.

Reverse-transcriptase PCR (RT-PCR) was performed using a SuperScript III OneStep RT-PCR system with Platinum Taq DNA polymerase (Life Technologies, Carlsbad, CA) following the manufacturer’s protocol. For RT-PCR the following thermal profile was used: a cDNA synthesis cycle of 30 min at 55°C, an initial denaturation of 94°C for 2 minutes, 40 cycles of 94°C for 15 sec, 55°C for 30 sec, and 68°C for 1 min, followed by a final extension of 68°C for 5 min. Separate reactions including ten micromolar concentrations of forward and reverse primers for each and every locus described above were performed. For a positive control, RT-PCR was performed with the primers AaDTr (CTGGATGGAGACGTAGAAGGC) and Df (CTCGATGCTCAAGCAGTACAT), which target *ACT* (VDAG_08445). Amplicons were visualized as above.

To verify the absence of genomic DNA in both of the RNA preparations, SuperScript III/RT Platinum Taq mix was omitted from PCR assays, and instead, two units of Platinum Taq DNA polymerase (Life Technologies, Carlsbad, CA) were used in reactions using the primers AaDTr and Df, in accordance with the manufacturer’s instructions.

### SELECTON analyses of positive and purifying selection in *MAT1-1-1*, *MAT1-2-1*, and other sex-related genes of *V. dahliae*

To test the hypothesis that *V. dahliae* mating type and meiosis-associated genes are being maintained under strong purifying selection, ratios of non-synonymous (amino-acid altering) to synonymous (silent) substitutions in *V. dahliae* genes (relative to sexual fungi) were calculated through the SELECTON server [66], [67]. All *MAT* genes used in this study were either identified directly through the FUNGIPath database, or were obtained through NCBI GenBank and verified as orthologs to either *MAT1-1-1* or *MAT1-2-1* using the ortholog search function in the FUNGIPath.

Additionally, ortholog search results from the FUNGIpath database from taxa within the subphylum *Pezizomycotina* were downloaded for 18 arbitrarily chosen, previously characterized genes associated with meiosis (Table S2), which represented a subset of the aforementioned 93 genes. Unaligned nucleotide sequences of *V. dahliae* orthologs and sequences identified through the FUNGIpath database [68] from at least nine other *Pezizomycotina* fungi were used as input to the SELECTON server, to provide the recommended number of sequences.

Selection pressure was estimated in the following 20 sex-related *V. dahliae* genes: *MAT1*–*1*–*1* and *MAT1*–*2*–*1* [28], [69]; the *RID* gene [36]; the nine *N. crassa* meiosis–specific genes *SPO11*, *SKI8*, *MUTL*, *RAD54*, *MSH4*, *MSH5*, *MUS50*, *RAD21,* and *REC8* [13]; the *N. crassa* gene *V-SNARE*, required for establishment and maintenance of sister chromatid cohesion [60]; and finally, seven *P. anserina* genes encoding pheromones, receptors, and genes related to pheromone biogenesis *STE24*, *RAM1*, *RCE1*, *KEX1*, *STE23*, *STE6,* and *PRE2* [63].

Nonsynonymous to synonymous substitution ratios (Ka/Ks) of *V*. *dahliae* genes were calculated using the SELECTON server [66], [67], based on alignments of *V. dahliae* genes with sequences from the following fungi with known sexual stages: *Aspergillus fumigatus* [70], *Aspergillus nidulans* [71], *Botrytis cinerea* [72], [73], *Chaetomium globosum* [74], *Colletotrichum graminicola* [75], [76], *Epichloë festucae* [77], *Eupenicillium crustaceum* [78], *Fusarium graminearum* [79], *Histoplasma capsulatum* [80], *Magnaporthe oryzae* [81], *Neurospora crassa* [61], *Nectria haematococca* [82], *Ophiostoma novo-ulmi* (NCBI GenBank ADB96163), *Penicillium chrysogenum* [83], *Podospora anserina* [65], *Sclerotinia sclerotiorum* [73], *Trichoderma reesei* [84] and *Zymoseptoria tritici* [85]. For each of the 20 *V. dahliae* genes analyzed, sequences from different taxa were used as input, based on availability. Transcript sequences of the relevant genes from fungal taxa were obtained from multiple sources, and accession numbers of fungal gene sequences are provided in Table 2 and Table S2.

Codon alignments were generated by the SELECTON server and for each codon, the Ka/Ks ratio was estimated using a Bayesian approach. SELECTON results for each codon were reported on a scale of 1–7, with scores of one or two indicating positive selection, and scores of six or seven indicating strong purifying selection. For comparative purposes, two evolutionary models with positive selection enabled were used in the analyses, namely the M8 model [86], [87] and the mechanistic–empirical combination (MEC) model [88]. SELECTON implements several codon models, each of which assumes different biological assumptions. The MEC model takes into account the differences between different amino-acid replacement probabilities. For analyses with the MEC model, eight categories for the distribution, a JTT empirical amino-acid matrix, and a high precision level were used. In cases where positive selection sites were detected using the MEC model, a likelihood ratio test between the results of the MEC model and the M8a (null) model was performed, by comparing Akaike Information Content (AIC) scores [89].

Estimates of selection in genes may be influenced by the choice of taxa used in the codon alignment. Therefore, for comparative purposes of the two mating type genes, Ka/Ks ratios within each of the other *MAT1-1-1* and *MAT1-2-1* sequences from other species were also calculated as above. For these analyses, the *V. dahliae* sequence was removed from the set of nucleotide sequences, and each sequence from every fungal species was considered independently as the query sequence. Thus, the Ka/Ks ratios of *MAT* loci were calculated for several sexual fungi, relative to the same set of taxa used to estimate selective pressures in *V. dahliae* mating type genes. Lastly, the Ka/Ks ratios in *MAT1-1-1* and *MAT1-2-1* from *Passalora fulvum*, a putatively asexual species, were calculated in comparison with the same set of sexual fungi used in the analyses of *V. dahliae* genes.

## Supporting Information

Figure S1 **Color-coded results of SELECTON analyses of 18** ***Verticillium dahliae*** **sex-related genes, compared to sequences from nine different sexual fungi in the** ***Pezizomycotina.***
(PPTX)

Table S1 ***V. dahliae*** **isolates used in this study along with country of origin, location, plant host, and mating types, as determined by PCR assays.**
(XLSX)

Table S2 **List of fungal gene sequence accessions and results from SELECTON analyses of** ***Verticillium dahliae g*** **enes associated with meiosis in model systems.**
(XLSX)
