# Supplementary material for: Maintenance of Sex-Related Genes and the Co-Occurrence of Both Mating Types in Verticillium dahliae
Source: PLoS One. 2014 Nov 10;9(11):e112145. doi: 10.1371/journal.pone.0112145 (PMC4226480; doi:10.1371/journal.pone.0112145)
Supplement: Figure S1 — Color-coded results of SELECTON analyses of 18 Verticillium dahliae sex-related genes, compared to sequences from nine different sexual fungi in the Pezizomycotina. (PPTX) [file pone.0112145.s001.pptx]

## Slide 1
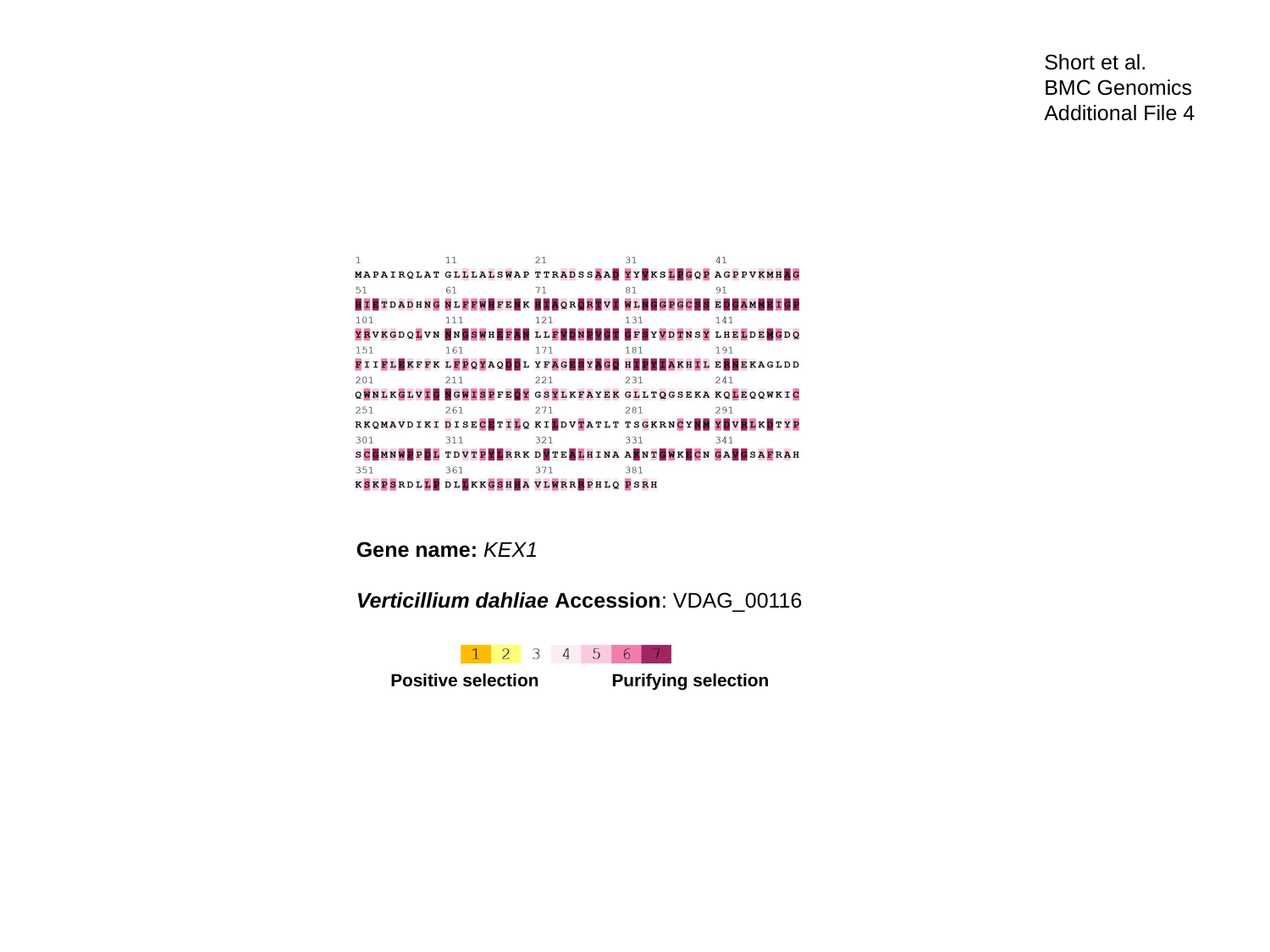

Short et al.
BMC Genomics
Additional File 4
Gene name: KEX1
Verticillium dahliae Accession: VDAG_00116
Positive selection Purifying selection

## Slide 2
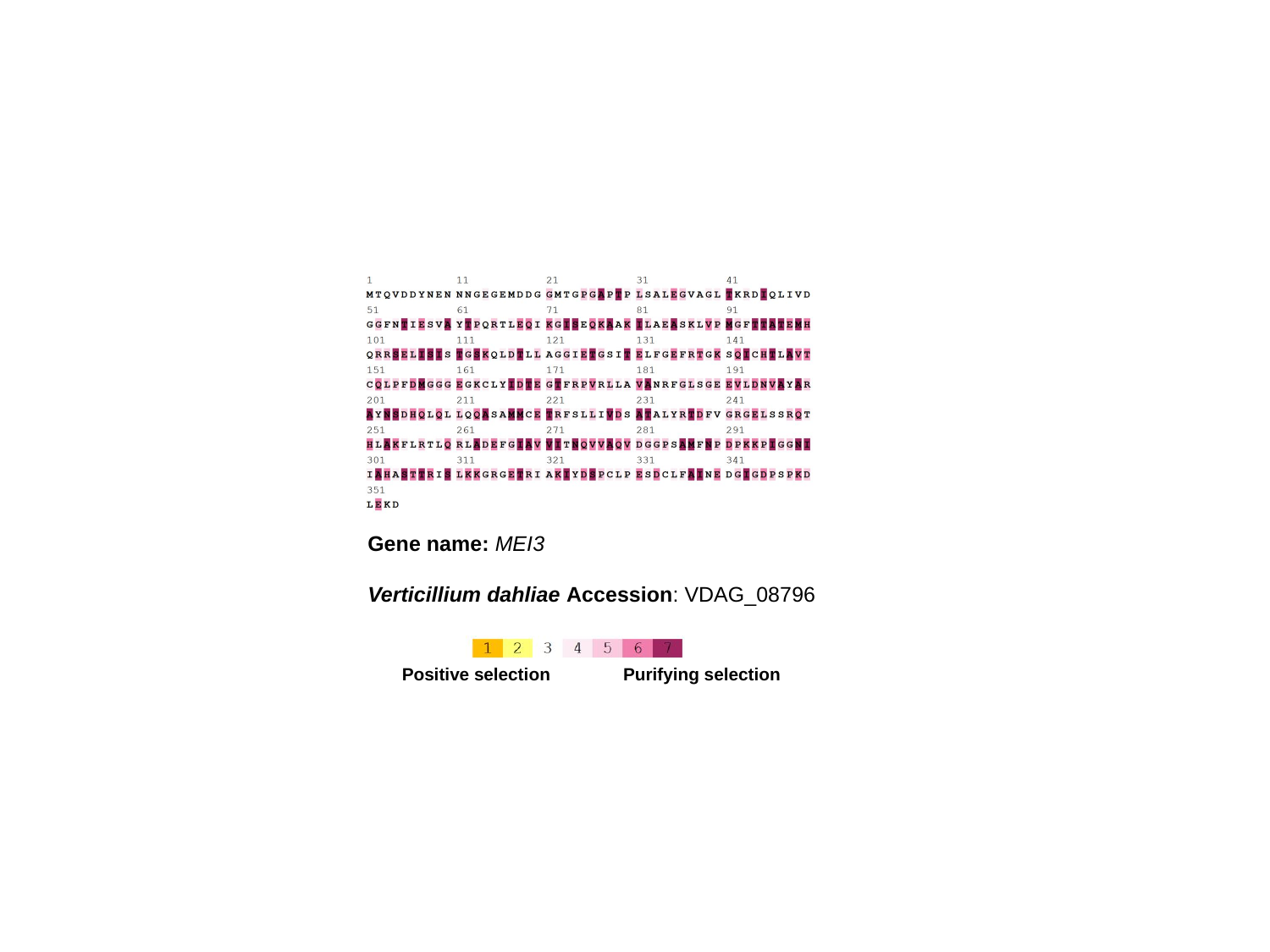

Gene name: MEI3
Verticillium dahliae Accession: VDAG_08796
Positive selection Purifying selection

## Slide 3
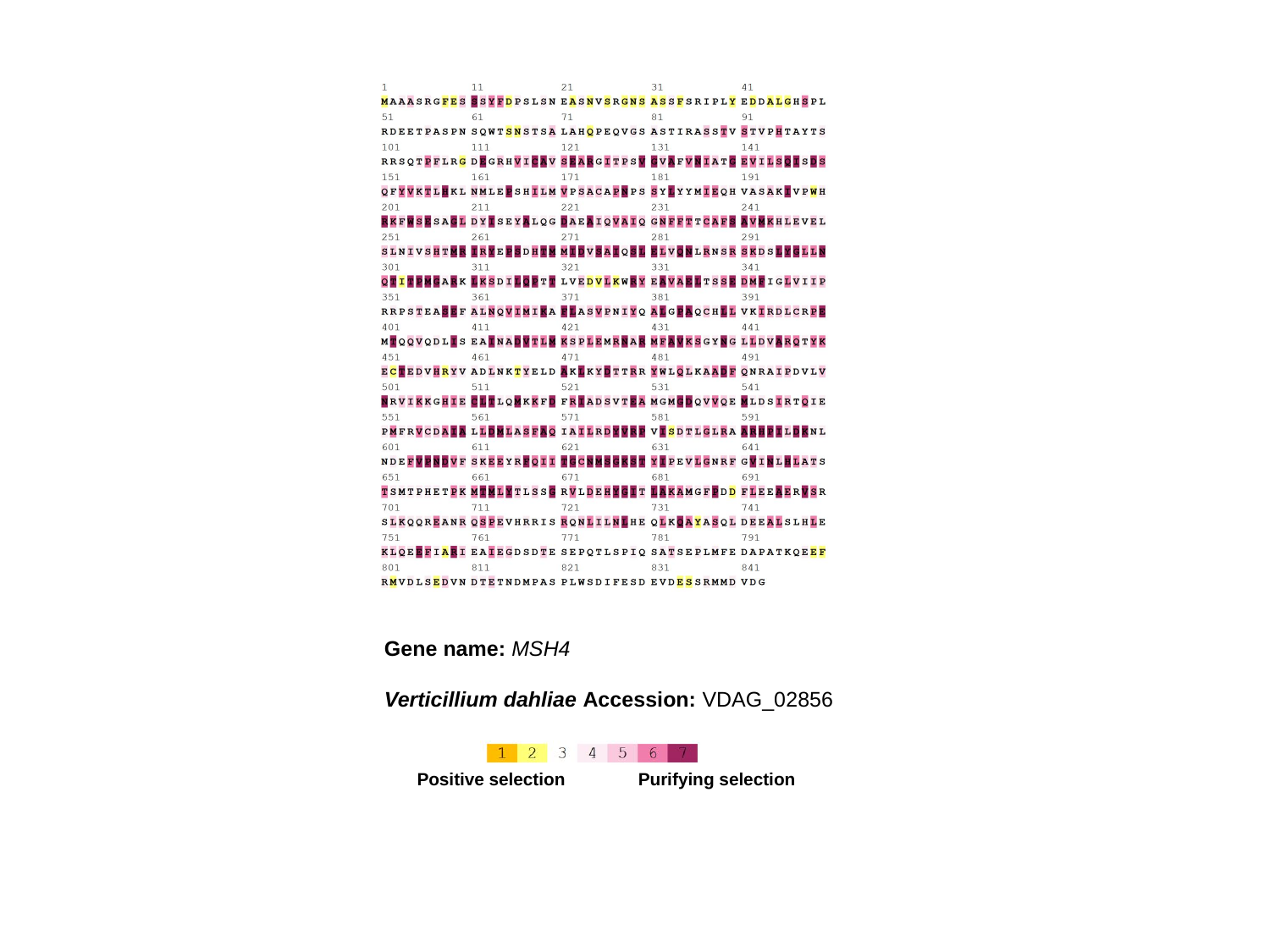

Gene name: MSH4
Verticillium dahliae Accession: VDAG_02856
Positive selection Purifying selection

## Slide 4
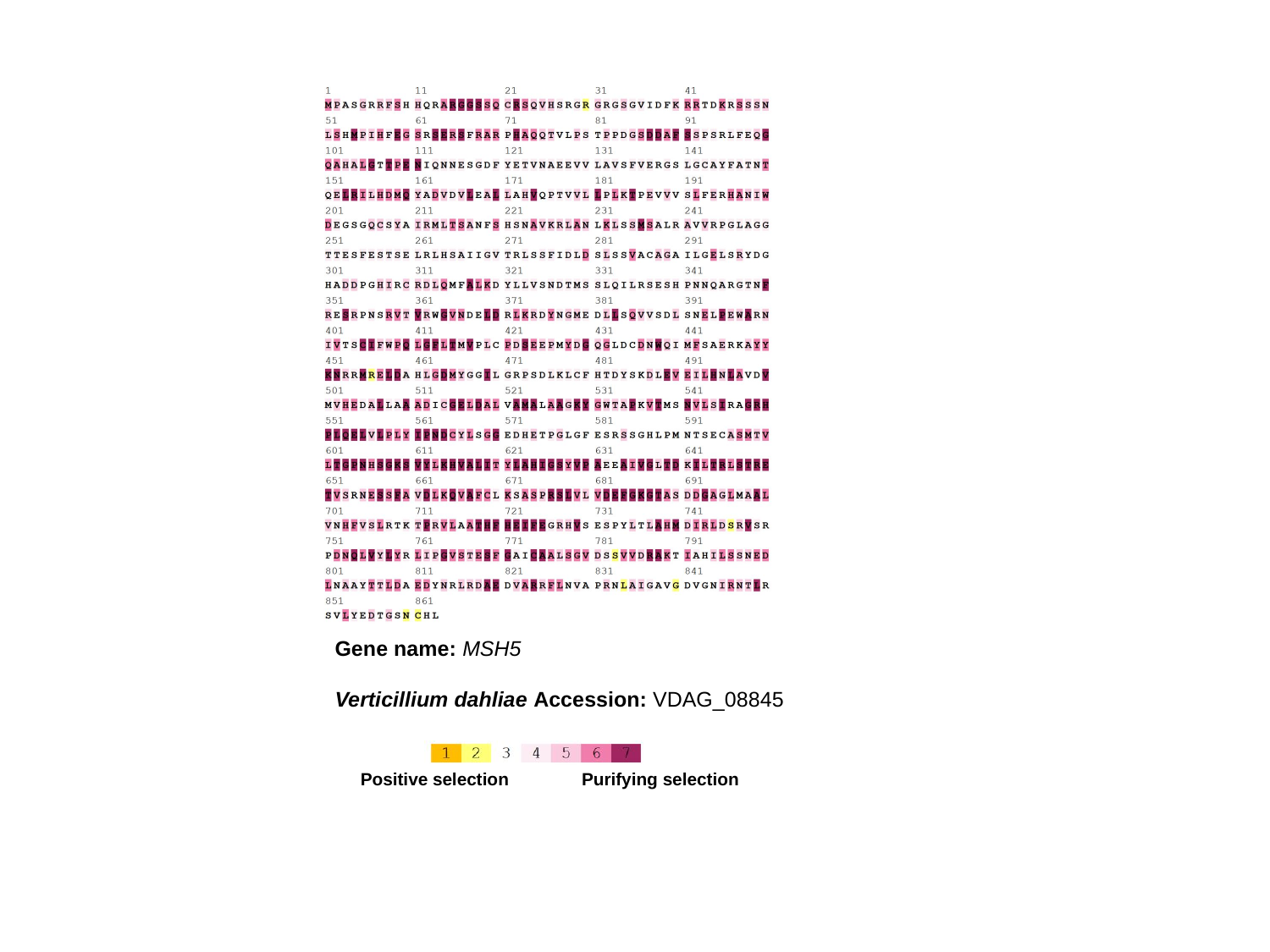

Gene name: MSH5
Verticillium dahliae Accession: VDAG_08845
Positive selection Purifying selection

## Slide 5
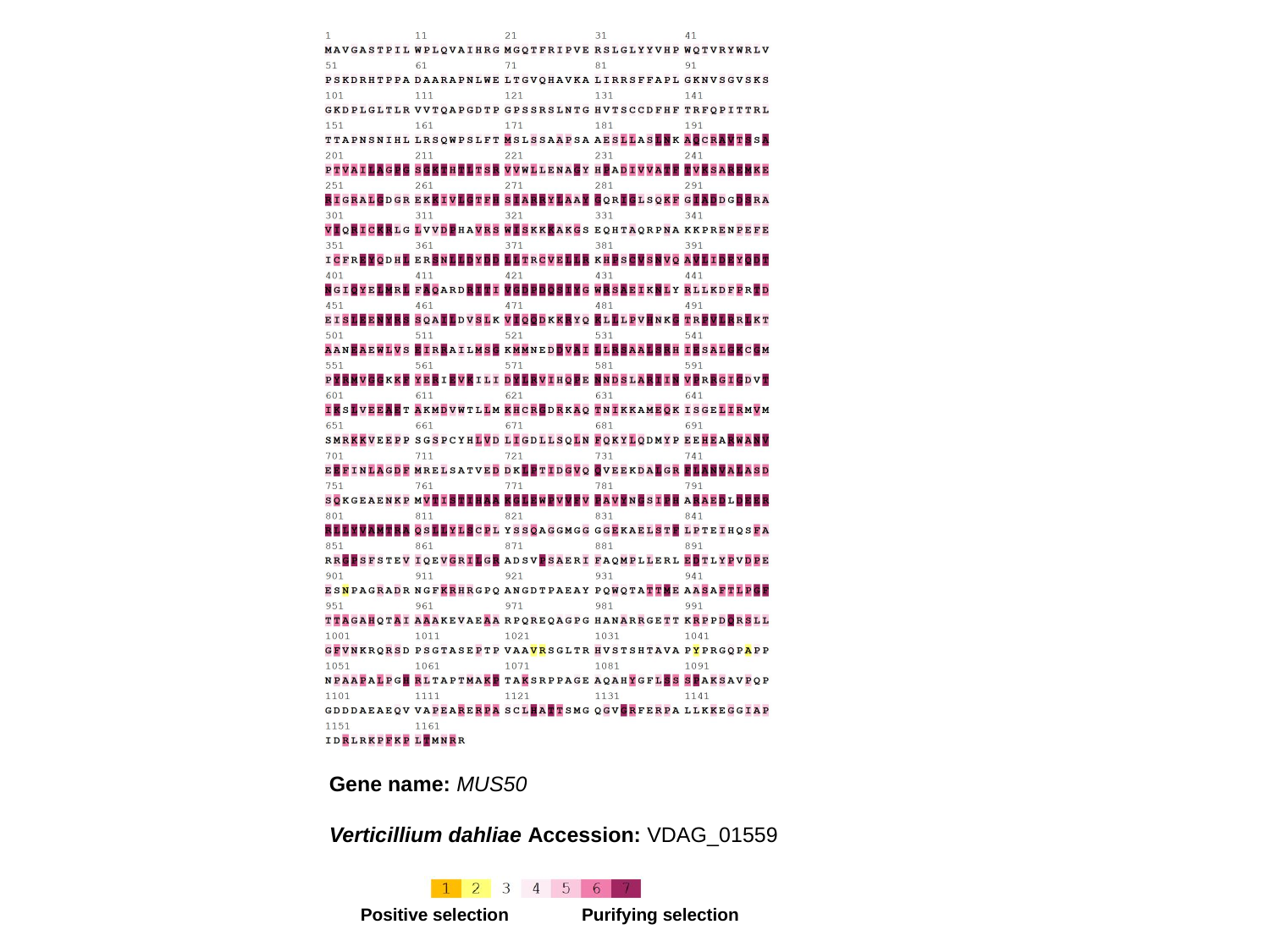

Gene name: MUS50
Verticillium dahliae Accession: VDAG_01559
Positive selection Purifying selection

## Slide 6
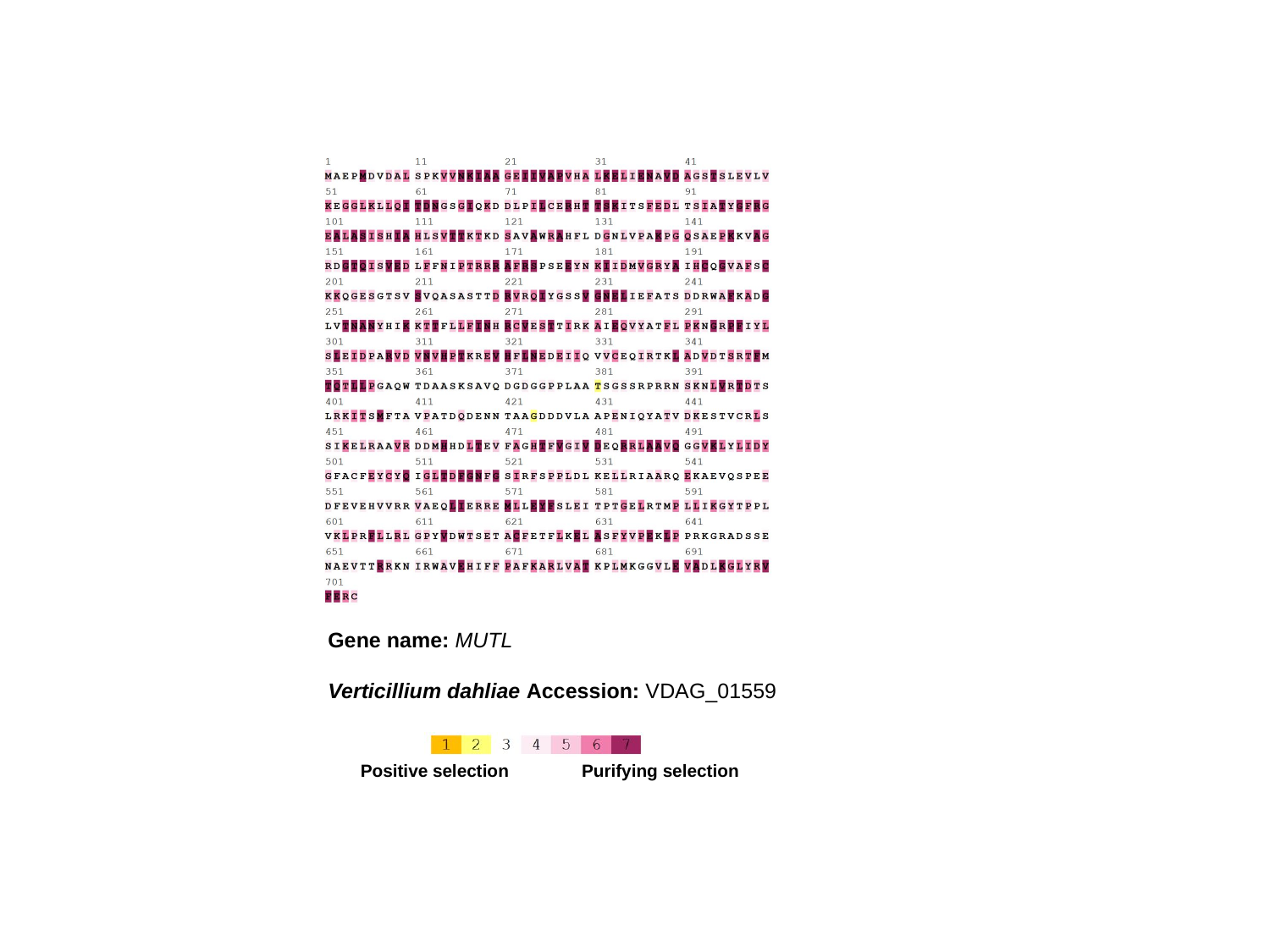

Gene name: MUTL
Verticillium dahliae Accession: VDAG_01559
Positive selection Purifying selection

## Slide 7
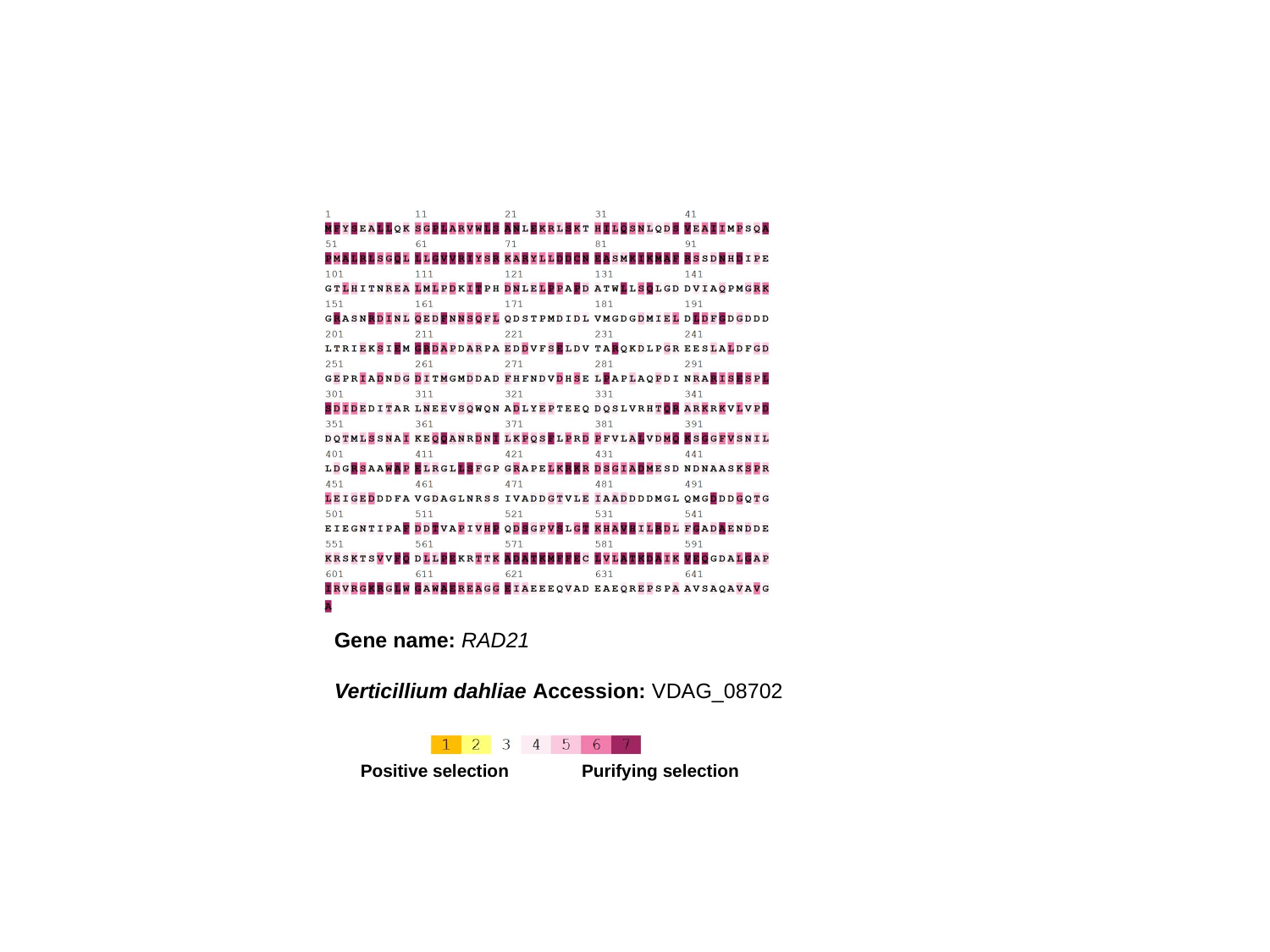

Gene name: RAD21
Verticillium dahliae Accession: VDAG_08702
Positive selection Purifying selection

## Slide 8
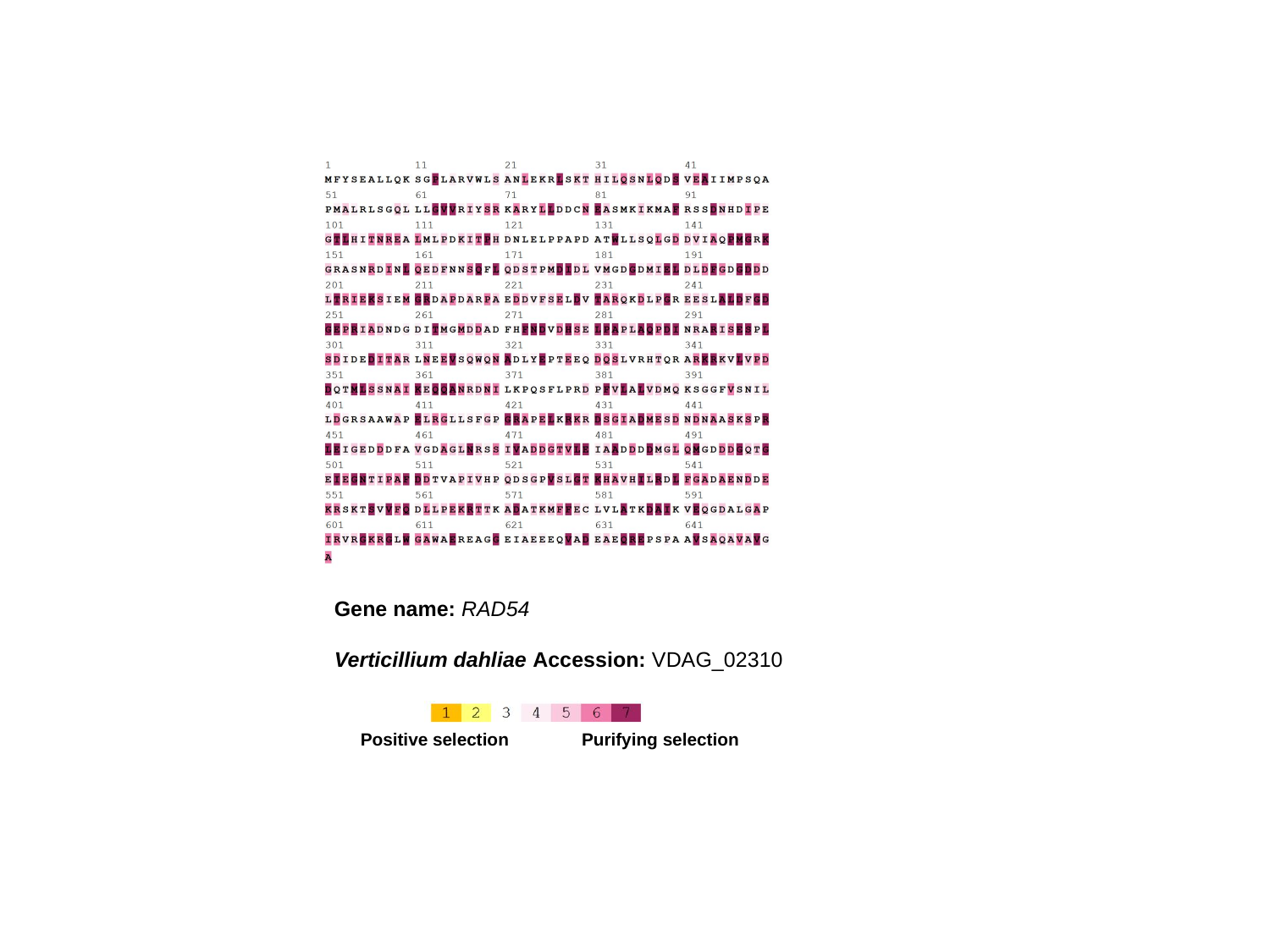

Gene name: RAD54
Verticillium dahliae Accession: VDAG_02310
Positive selection Purifying selection

## Slide 9
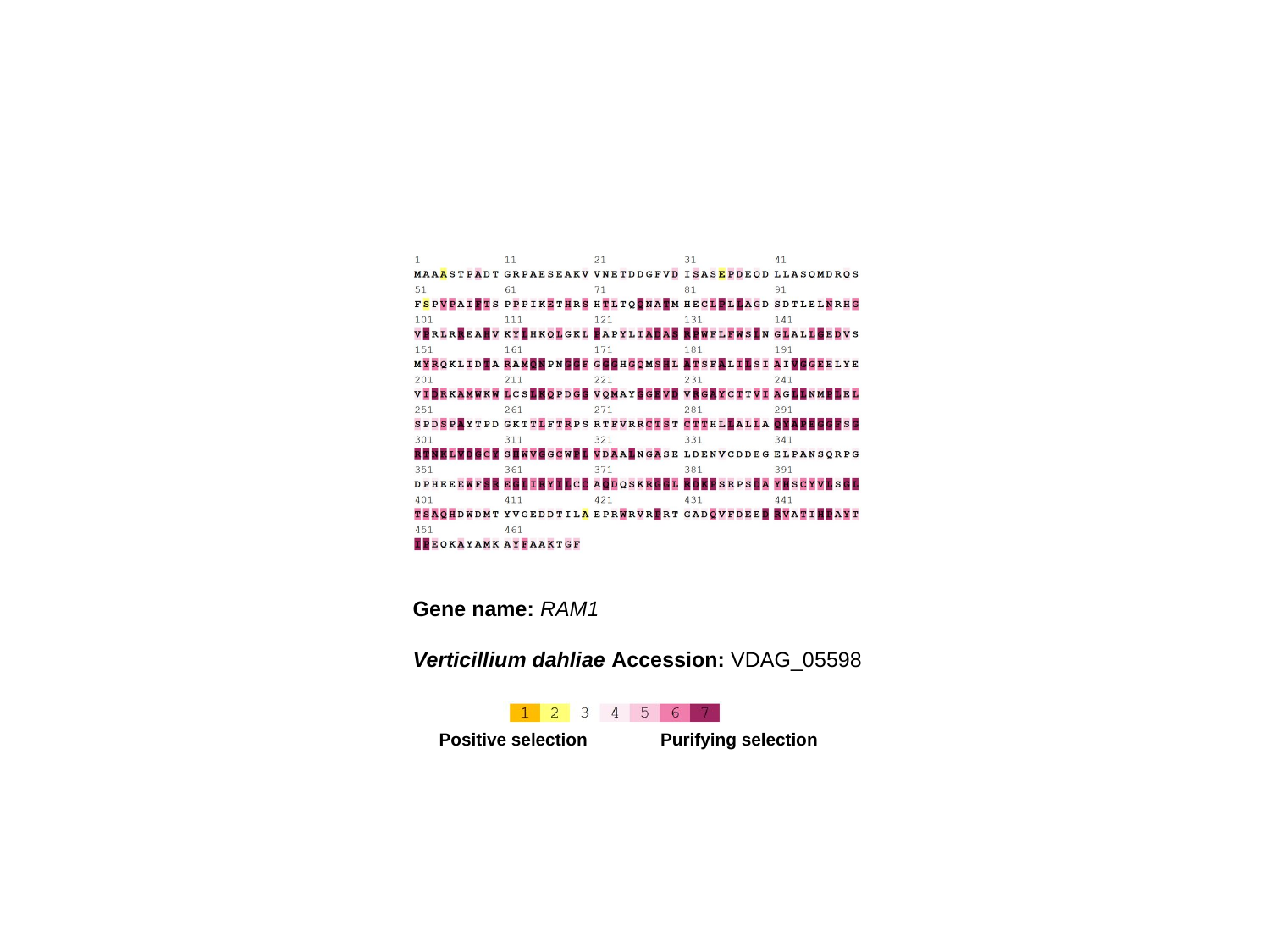

Gene name: RAM1
Verticillium dahliae Accession: VDAG_05598
Positive selection Purifying selection

## Slide 10
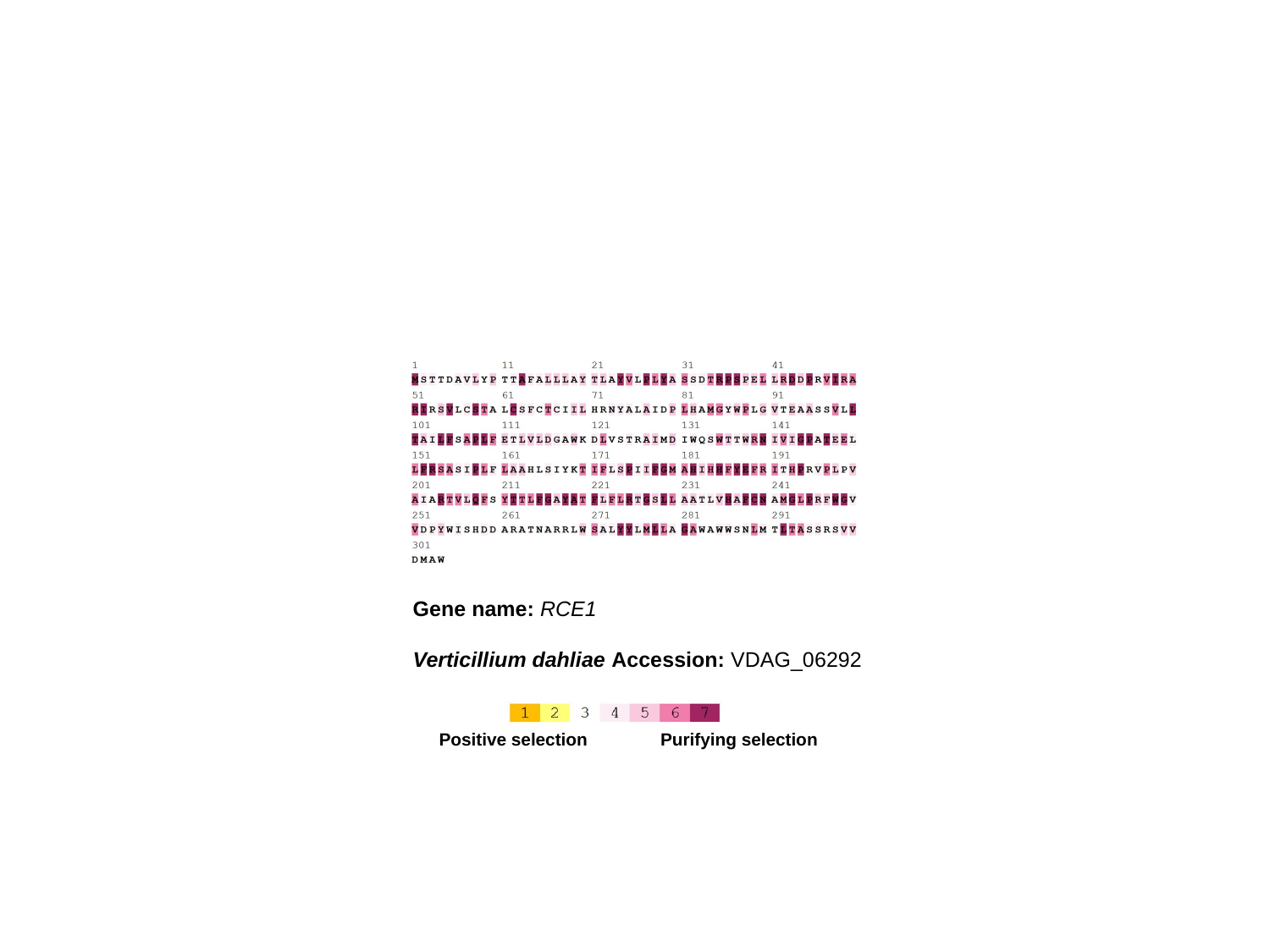

Gene name: RCE1
Verticillium dahliae Accession: VDAG_06292
Positive selection Purifying selection

## Slide 11
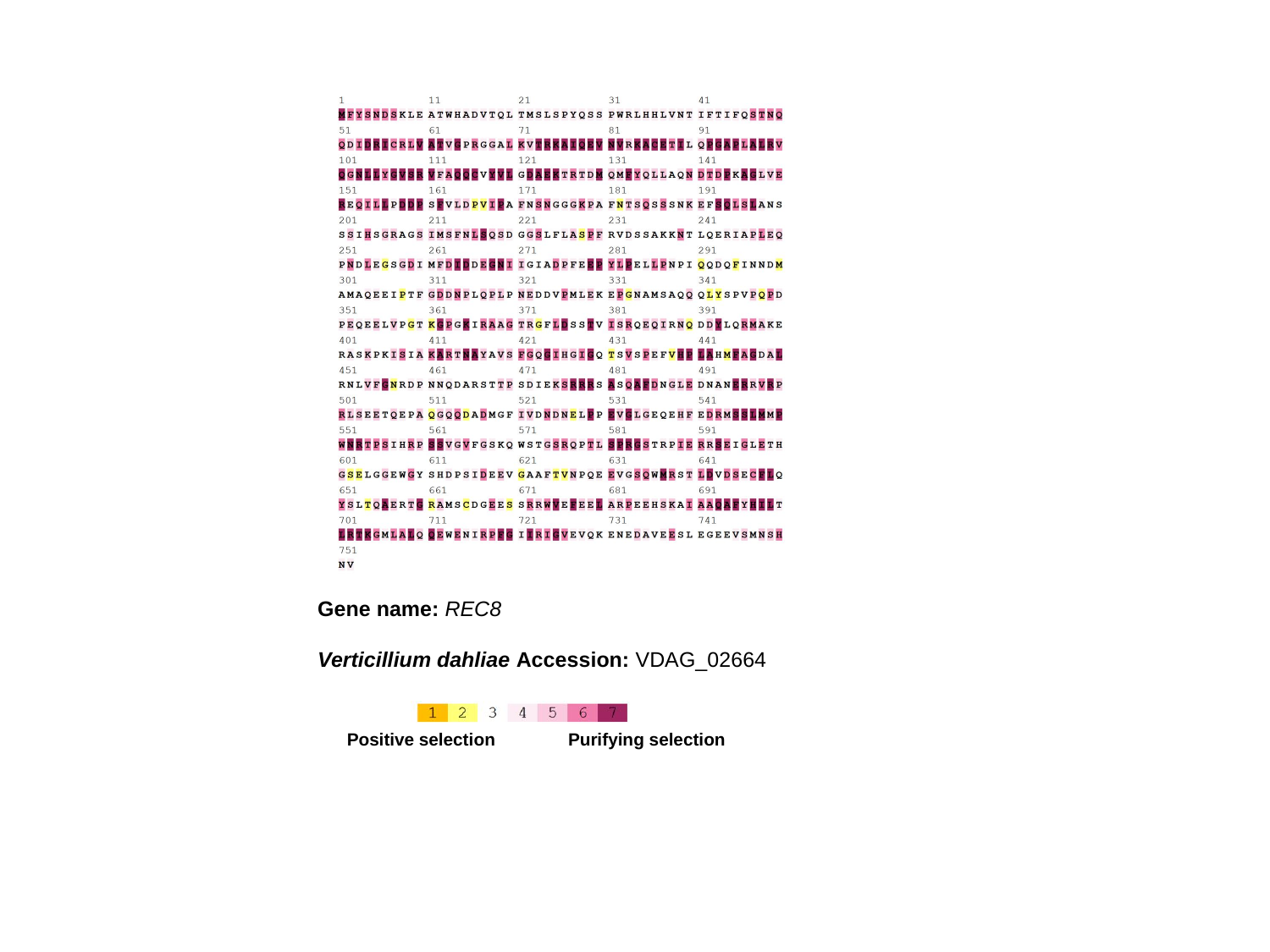

Gene name: REC8
Verticillium dahliae Accession: VDAG_02664
Positive selection Purifying selection

## Slide 12
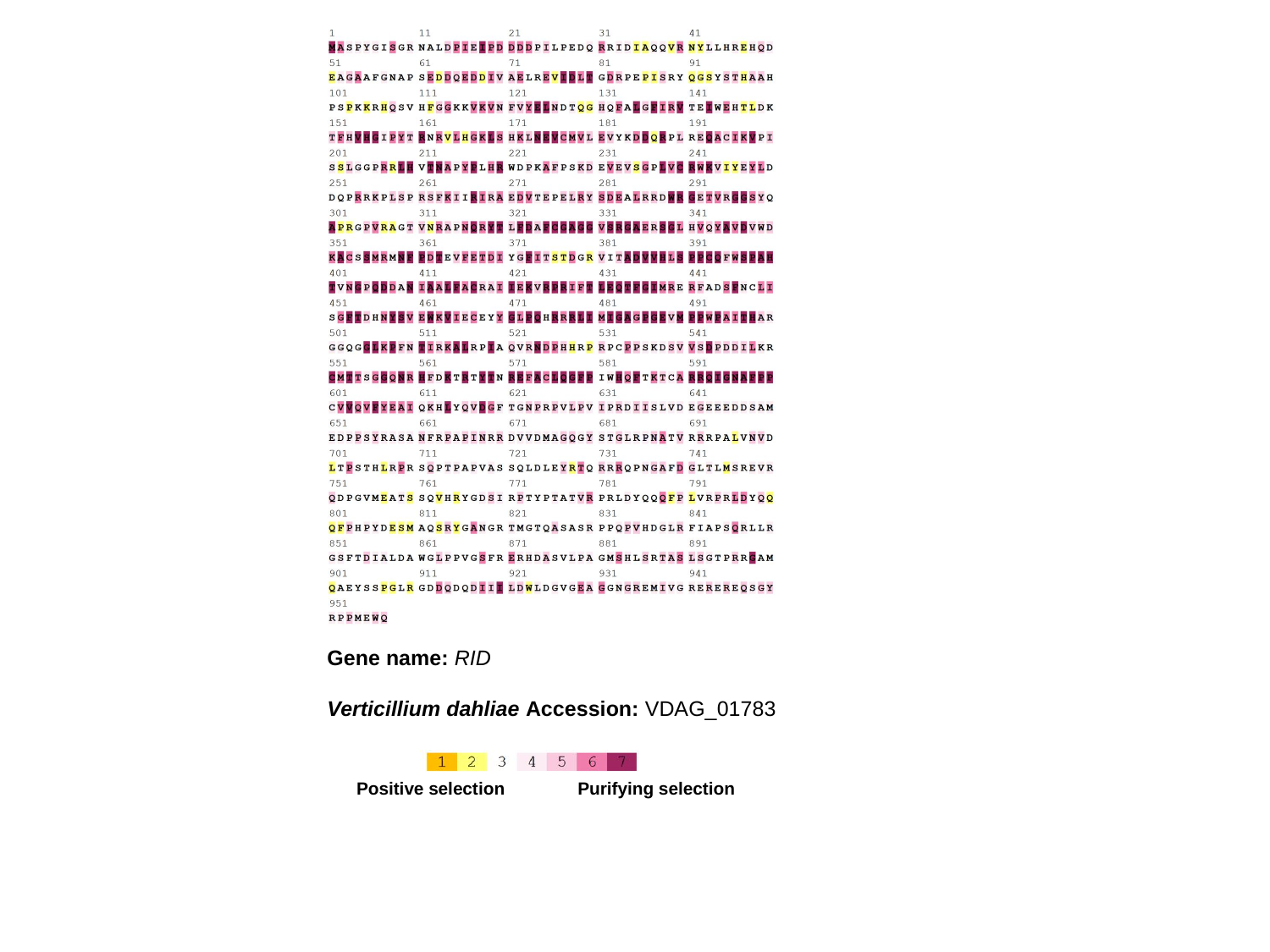

Gene name: RID
Verticillium dahliae Accession: VDAG_01783
Positive selection Purifying selection

## Slide 13
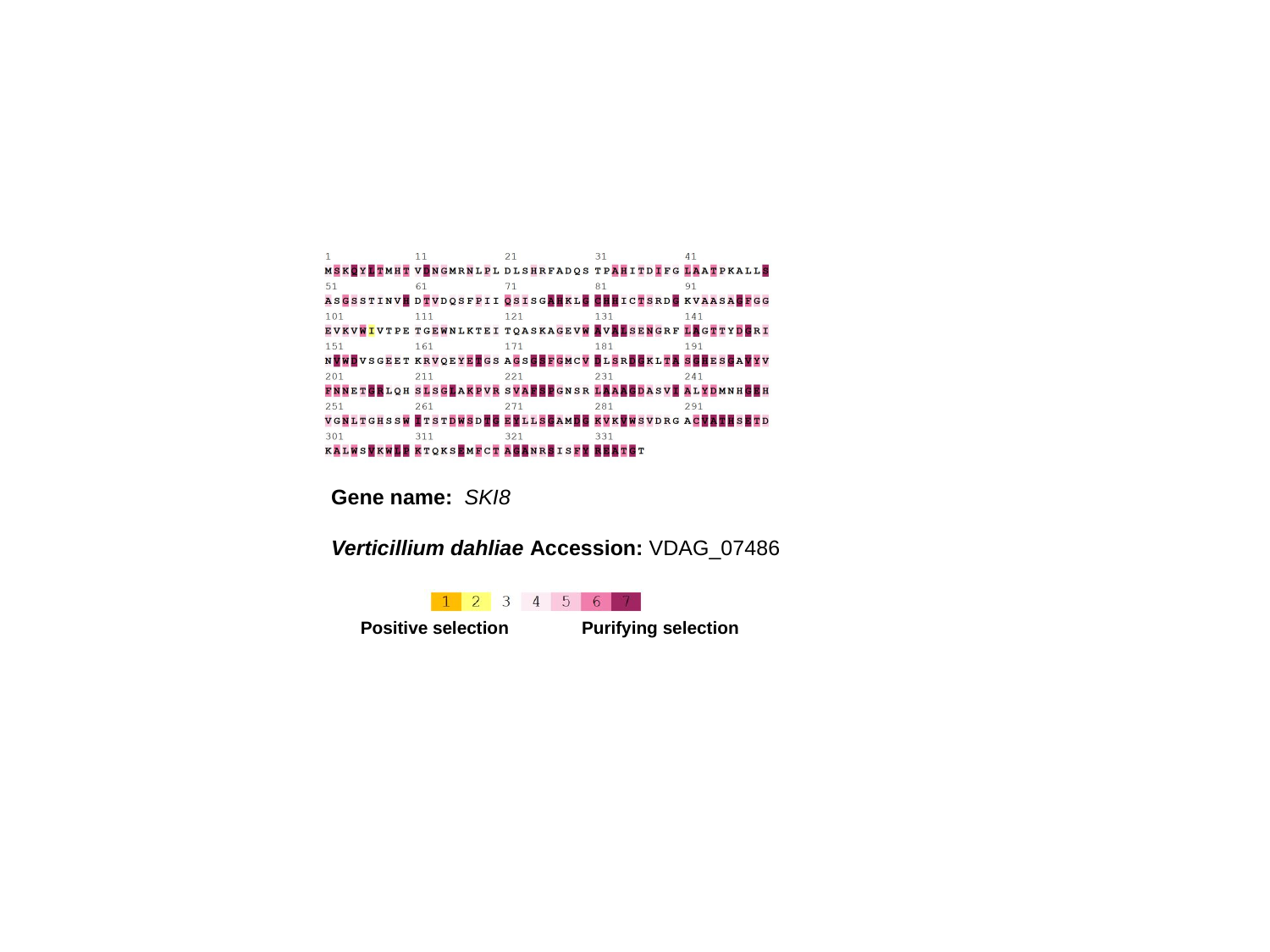

Gene name: SKI8
Verticillium dahliae Accession: VDAG_07486
Positive selection Purifying selection

## Slide 14
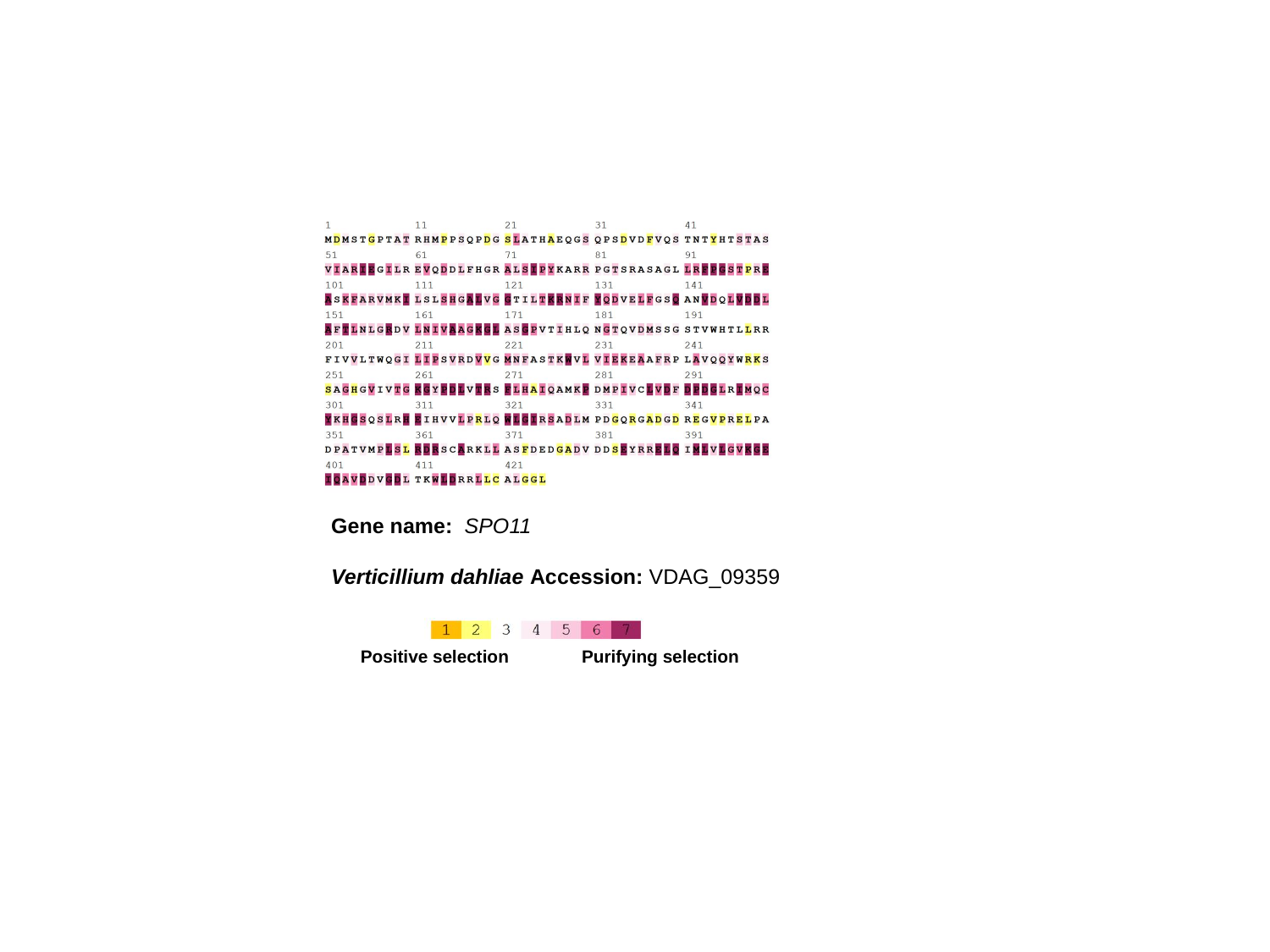

Gene name: SPO11
Verticillium dahliae Accession: VDAG_09359
Positive selection Purifying selection

## Slide 15
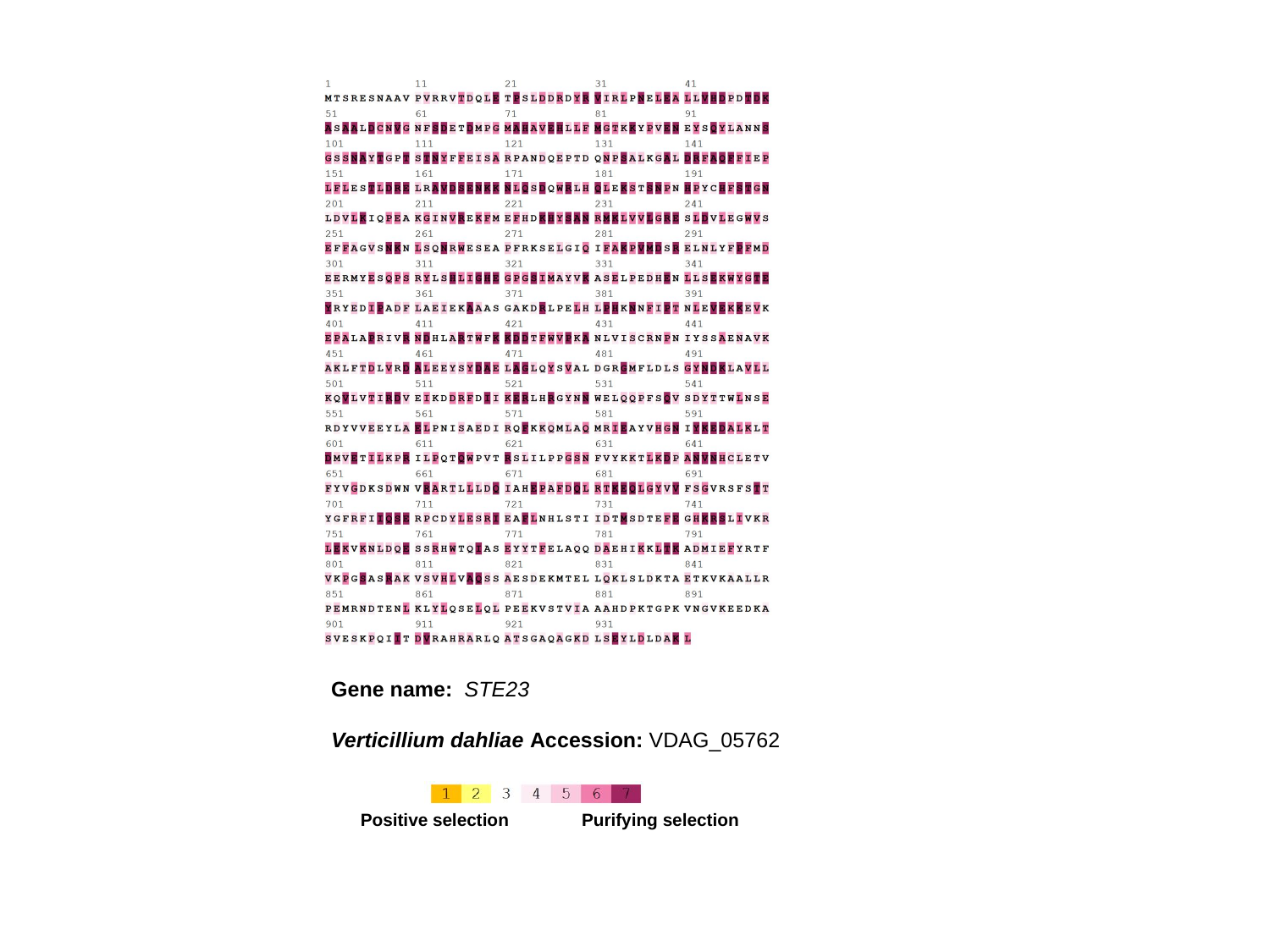

Gene name: STE23
Verticillium dahliae Accession: VDAG_05762
Positive selection Purifying selection

## Slide 16
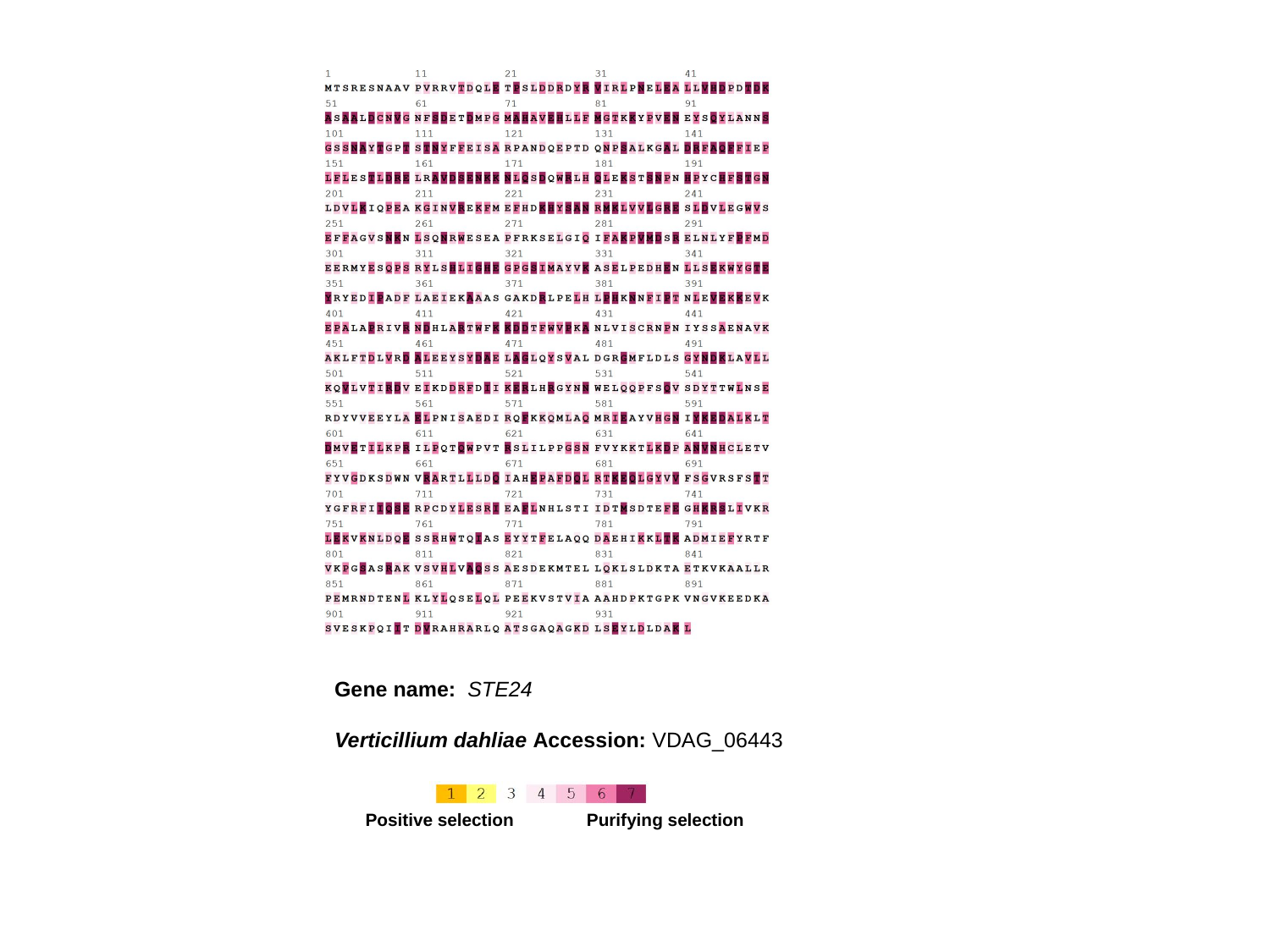

Gene name: STE24
Verticillium dahliae Accession: VDAG_06443
Positive selection Purifying selection

## Slide 17
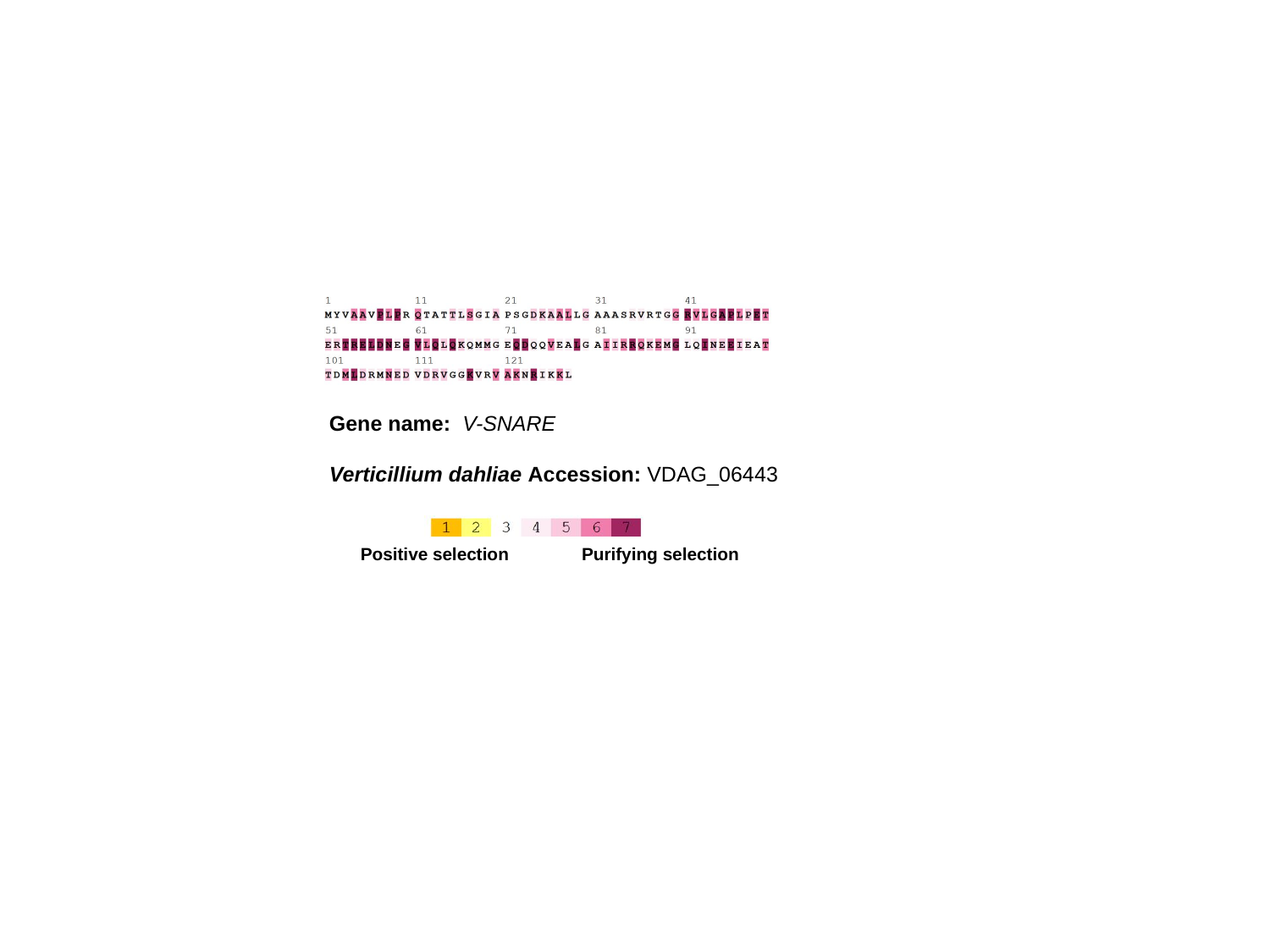

Gene name: V-SNARE
Verticillium dahliae Accession: VDAG_06443
Positive selection Purifying selection

## Slide 18
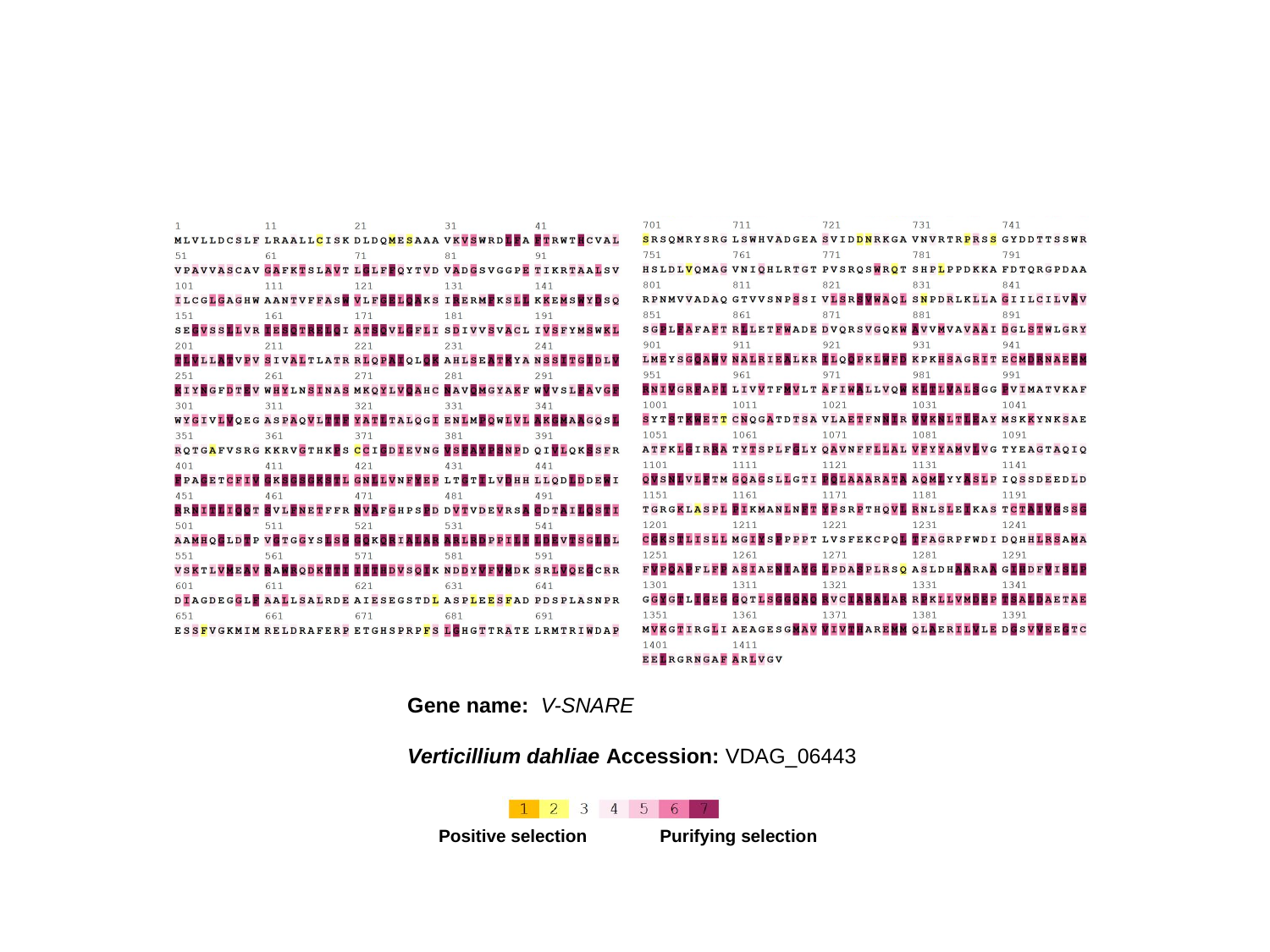

Gene name: V-SNARE
Verticillium dahliae Accession: VDAG_06443
Positive selection Purifying selection
